# The predicted transcription factors of the immune-related genes in Pacific white shrimp (*Litopenaeus vannamei*) respond to *Fusarium solani* infection

**DOI:** 10.1016/j.cirep.2026.200288

**Published:** 2026-05-03

**Authors:** Yusuf Jibril Habib, Akram Ismael Shehata, Chengjie Yao, Haifu Wan, Jiaming Lin, Hui Ge, Mohammed F. El Basuini, Mayada Alhoshy, Yilei Wang, Ziping Zhang

**Affiliations:** a Department of Medical Analysis, Faculty of Applied Science, Tishk International University, Erbil, Iraq; b Department of Animal and Fish Production, Faculty of Agriculture (Saba Basha), Alexandria University, Alexandria, 21531, Egypt; c College of Fisheries, Jimei University, Xiamen, Fujian 361021, China; d Key Laboratory of Cultivation and High-value Utilization of Marine Organisms in Fujian Province, Fisheries Research Institute of Fujian, Xiamen 361012, China; e Faculty of Agriculture, Tanta University, Tanta 31527, Egypt; f College of Marine Science, Fujian Agriculture and Forestry University, Fuzhou, Fujian 350002, China; g Key Laboratory of Marine Biotechnology of Fujian Province, Institute of Oceanology, Fujian Agriculture and Forestry University, Fuzhou, 350002, PR China

**Keywords:** Transcription factors, Immune-related genes, Pacific white shrimp, Gene Regulation, *Fusarium solani*

## Abstract

Aquatic organisms need immune-related genes to fight infections. In response to pathogenic challenges, transcription factors activate or repress immune-related genes at the right time and in the right amounts. In this study, we will identify and describe the transcription factors that govern immune-related genes in Pacific white shrimp (*Litopenaeus vannamei*) and examine how they react to *Fusarium solani*, a pathogenic fungus. We found immune-related genes in shrimp using genomic data and TBLASTN with a 1e-5 E-value cutoff. ORF Finder and Smart-BLAST showed gene identities and predicted protein characteristics. Gene structure, motifs, and transcription factor families were analysed with GSDS, MEME, and promoter prediction tools. At six time points, healthy shrimp were challenged with *F. solani* and sampled from hemocytes, gills, and hepatopancreas. RNA was extracted, and qRT-PCR measured gene expression, normalised to ef1-α, using the 2^−ΔΔCT^ method. A total of 21 immune-related genes were identified in the L. *vannamei* genome through genome-wide analysis. Promoter analysis of the 2000 bp upstream regions of these genes revealed 4167 predicted transcription factors binding events across the complete gene set, with *C/EBP, GATA, Octamer, TBP, and Sp1* being the most persistent. Four transcription factors (*C/EBPγ, GATA4, SOX-1*, and *SOX-2*) were detected in all three healthy L. *vannamei* organs (haemocytes, hepatopancreas, and gills). Compared to the control group, all four TF gene expressions varied significantly in response to infection at distinct time periods post-infection. We propose that fungal infections may activate TFs. This study will help aquaculture systems manage fungal infections by improving our understanding of Pacific white shrimp immune responses' molecular and genetic basis.

## Highlights

• Identified 21 immune-related genes in *Litopenaeus vannamei*.
• Predicted 4167 TF family members regulating immune genes.
• C/EBP, GATA, Oct, TBP, and Sp1 were the most enriched TFs.
• Fungal challenge altered TF expression across immune tissues.
• Reveals regulatory basis of antifungal immunity in shrimp.

## Introduction

Among commercially farmed penaeid species, *Litopenaeus vannamei* (Pacific white shrimp) occupies a uniquely dominant position in the global aquaculture industry. Its exceptional growth rate, broad salinity tolerance, high fecundity, and favorable feed-conversion efficiency have collectively driven its adoption as the principal cultivated shrimp species across Asia, Latin America, and, increasingly, parts of the Middle East and Africa [[1], [2], [3], [4]]. Global production trends clearly reflect this development, as the species now represents the largest share of total penaeid production worldwide, with output increasing dramatically over the past two decades [[1], [2], [3], [4]]. This expansion has, however, been accompanied by an intensification of farming practices, including elevated stocking densities, reduced water exchange, and increased reliance on formulated diets, conditions that, while economically motivated, have simultaneously created environments conducive to pathogen proliferation and disease outbreaks. As a result, infectious disease has emerged as the most persistent and economically damaging constraint on the continued expansion of L. *vannamei* aquaculture [5].

The spectrum of pathogens threatening farmed shrimp is broad and well-documented. Viral agents, most notably white spot syndrome virus (WSSV) and yellow head virus (YHV), have historically been responsible for catastrophic production losses, decimating pond populations within days of outbreak [5]. Bacterial pathogens, particularly members of the genus *Vibrio*, including *Vibrio alginolyticus, Vibrio anguillarum, V. harveyi*, and *V. parahaemolyticus*, are consistently implicated in acute hepatopancreatic necrosis disease and other vibriosis syndromes that compromise larval and juvenile survival [5]. Against this backdrop, fungal pathogens have emerged as a distinct and increasingly recognized category of infectious threat. *Fusarium solani* has drawn particular attention as the etiological agent of fusariosis (black spot disease) in penaeid shrimp, a condition characterized by progressive cuticular melanization, tissue invasion, and elevated mortality in both juvenile and adult populations [1,6,7]. Unlike viral and bacterial pathogens, *F. solani* infections have received comparatively limited scientific scrutiny, creating a knowledge gap that is increasingly difficult to justify given the pathogen's growing prevalence in intensive aquaculture systems. The widespread and often indiscriminate use of antimicrobial agents to manage bacterial co-infections further complicates the situation, as antibiotic resistance acquisition and environmental contamination limit the sustainability of chemotherapy-based management approaches [1,6,7]. This underscores the urgent need to understand the host-side molecular mechanisms that govern resistance to fungal infection, which may ultimately underpin the development of biologically informed and durable disease control strategies.

Unlike vertebrates, which possess both innate and adaptive arms of immunity, shrimp and other crustaceans rely exclusively on innate immune mechanisms for pathogen recognition, containment, and elimination. Hemocytes serve as the functional cornerstone of this system, mediating a diverse array of defensive responses, including phagocytosis, encapsulation, nodule formation, and the synthesis and release of humoral factors [8,9]. Pathogen recognition is initiated when hemocyte-associated pattern recognition receptors (PRRs) detect conserved pathogen-associated molecular patterns (PAMPs), structural motifs such as lipopolysaccharides, peptidoglycans, and fungal β−1,3-glucans that are absent from host cells but broadly conserved across classes of microorganisms [8,9]. PRR activation rapidly starts a series of signalling processes that lead to several important immune defense pathways. These include the prophenoloxidase (proPO) activation cascade, which produces toxic melanin-related compounds at infection sites; antioxidant defense systems that remove reactive oxygen species generated during the oxidative burst; and the production of antimicrobial peptides (AMPs), which directly damage microbial cell membranes [8,9]. These responses are not constantly active but are carefully controlled at the gene expression level. Exposure to pathogens causes rapid and coordinated changes in the expression of immune-related genes in target tissues, including hemocytes, gills, and the hepatopancreas [7].

The development of transcriptomic and high-throughput sequencing technologies has greatly improved the identification of differentially expressed immune genes in *Litopenaeus vannamei* exposed to bacterial, viral, and environmental stressors [10], providing detailed molecular insights into the host response under different pathogenic conditions [11,12]. However, despite the growing amount of transcriptomic data, the upstream transcriptional mechanisms that control these changes in immune gene expression, especially during fungal infection, remain poorly understood.

Transcription factors (TFs) are DNA-binding proteins that recognize specific sequences and play a central role in controlling gene expression in eukaryotic organisms. As one of the oldest and most evolutionarily conserved protein families, TFs have been identified across almost all eukaryotic lineages and are considered essential regulators of development, cellular balance, and adaptive stress responses [13]. Their function depends on binding to short and flexible DNA sequences, known as cis-regulatory elements or transcription factor binding sites (TFBS), which are mainly located in gene promoter regions [14]. Because these binding sequences are often variable, a single TF can regulate a wide range of genes, while interactions among multiple TF families allow highly precise and gene-specific regulation [15]. In practical terms, this means that the same group of TFs can produce different patterns of gene expression depending on the combination of interacting factors, the accessibility of chromatin at target sites, and the cellular environment in which they act [14,15]. In immune biology, TFs act as the molecular link between external pathogen signals and the nuclear machinery responsible for gene transcription, converting upstream PRR signalling into coordinated and time-dependent activation or repression of immune genes. Therefore, identifying the TF families that regulate immune gene promoters is essential for understanding how shrimp initiate, maintain, and resolve immune responses at the molecular level.

Although significant progress has been made in identifying immune-related genes in L. *vannamei* through transcriptomic studies, the regulatory networks that connect transcription factors (TFs) to specific immune gene promoters are still not fully understood in this species. Most previous studies have mainly focused on TF-mediated regulation of immune responses to bacterial and viral infections, where factors such as NF-κB, AP-1, and components of the Toll pathway have been identified as major regulators [11,12]. In contrast, the transcriptional regulatory mechanisms involved in the host response to fungal infection in penaeid shrimp remain largely unknown. *Fusarium solani*, despite its known pathogenic effects and association with mortality in cultured L. *vannamei* populations [1,6], has not previously been investigated at the TF level in this host. This knowledge gap is important because, without identifying which TF families regulate immune genes during fungal infection, it is difficult to fully understand the molecular basis of the host immune response or identify effective targets for disease control. Addressing this gap requires an integrated approach that combines genome-wide computational prediction of TF binding sites with experimental validation of TF expression patterns under controlled infection conditions, which is the strategy used in the present study.

Accordingly, the present study was designed with two related objectives. First, to systematically identify and characterize the TF families predicted to regulate a selected set of 21 immune-related genes in the L. *vannamei* genome through genome-wide promoter analysis. Second, to experimentally examine the expression patterns of four candidate TFs [*C/EBPγ, GATA4, SOX-1*, and *SOX-2*] in three major immune tissues (hemocytes, hepatopancreas, and gills) at different time points after experimental challenge with *Fusarium solani*. By combining silico promoter prediction with quantitative gene expression analysis, this study aims to clarify the transcriptional regulatory framework underlying the shrimp immune response to fungal infection. We hypothesized that the TF families most frequently predicted in immune gene promoters, particularly C/EBP and GATA, would show significant tissue-specific upregulation after fungal challenge, suggesting their active role in regulating the host transcriptional response to *F. solani*. To the best of our knowledge, this is the first study to combine genome-wide TF binding site prediction with in vivo expression profiling following fungal infection in L. *vannamei*, and its findings are expected to improve the mechanistic understanding of crustacean antifungal immunity while providing a molecular basis for future disease management strategies in penaeid aquaculture.

## Materials and methods

### Identification of immune-related genes in the genome of L. *vannamei*

To find the immune-related genes expressed in the immune tissues of decapod crustaceans, we searched the L. *vannamei* genome (https://www.ncbi.nlm.nih.gov/genome QCYY00000000.1). We obtained the query sequences for similarity searches from publicly available databases, such as NCBI GenBank, based on prior annotations and their relevance to the target gene family. We employed the NCBI BLAST suite's BLASTp and TBLASTN algorithms to identify homologous sequences within the target dataset, using these reference sequences as queries. To ensure that the identified sequences were reliable and biologically pertinent, we applied several filtering criteria. We used an E-value threshold of ≤ 1 × 10⁻⁵ to filter out initial hits that were likely to happen by chance. Also, only sequences with a minimum percent identity of 30% and an alignment coverage of 70% (compared to the length of the query) were kept. For TBLASTN searches, the alignment coverage was based on the translated nucleotide region that matched the query protein. Following a manual review, we removed redundant sequences and partial fragments that lacked significant conserved domains. When there were more than one isoform or very similar sequences, only the longest and most complete representative sequence was kept for later analyses. To ensure that the candidate sequences were correct, we used domain prediction tools such as Pfam and SMART to check that the conserved domain architecture was consistent with what we already knew about the gene family. These criteria were chosen to strike a balance between sensitivity and specificity, which means they can find real homologs while reducing the number of false-positive matches. The PI/MW tool (http://web.expasy.org/protparam/) were used to predict the putative molecular weight (MW) and isoelectric point (PI) of the candidate protein. Using Wolfpsort (https://wolfpsort.hgc.jp/) to predict their subcellular localization.

### Gene structure, subcellular localization, and conserved motifs analysis

The gene structure of all candidate genes was predicted using the Gene Structure Display Server 2.0 (GSDs) by aligning the cDNA sequences with the corresponding genomic DNA sequence from the same genes (http://gsds.cbi.pku.edu.cn/). The MEME online program analyzed the conserved motifs in proteins using the following parameters: any number of repetitions, a maximum of 20, and an optimum motif width of 30–70 residues (http://meme-suite.org/tools/meme).

### Immune-related gene transcription factor family prediction

Transcription factor binding sites (TFBS) within the promoter regions were predicted with Alibaba 2.1. This technology, mostly focused on vertebrate transcription factor binding patterns, was utilised here as a preliminary screening method to uncover potential regulatory motifs. A multitude of transcription factors and their core binding sequences are evolutionarily conserved across taxa, including arthropods, facilitating first cross-species inferences. Nonetheless, owing to potential lineage-specific variations in transcription factor binding affinities, these predictions must be regarded with caution and deemed speculative. Additional experimental validation is necessary to ascertain their functional significance. The transcription factor family for the identified immune-related genes was predicted by analyzing the 2000 bp upstream sequence of each gene using the CDS sequence to map it into the genome sequence. This analysis provided us with information about the promoter of each gene. The online promoter 2.0 prediction servers (http://www.cbs.dtu.dk/services/promoter/) and Alibaba 2.1 (http://www.gene-regulation.com/pub/programs/alibaba2/index.html) were used to predict the transcription factor binding sites at 2000 bp upstream of the transcription start site (TSS) [16].

### An analysis of transcription factor expression patterns

The whole genome database (NC-009,626.1 ASM378908v1) of Pacific white shrimp was searched using human, fruit fly, and mouse TFs as queries to identify the expression profiling of four predicted transcription factors (*GATA4 LOC113820974, C/EBP LOC113814445, SOX-1 LOC113829620*, and *SOX-2 LOC113828508*). All their amino acid sequences were obtained from the NCBI database (http://www.ncbi.nlm.nih.gov/). We used these as BLASTP queries to search against the protein sequence of the L. *vannamei* genome, using an E-value cut-off of 1e-10. The accuracy of the candidate genes was confirmed by conducting a reciprocal BLAST search using the candidate L. *vannamei* TFs gene sequence as a query.

### Experimental animals, fungal challenge experiment and sampling

#### Experimental animals

The fungal challenge experiment was conducted on experimental animal selection and sample procedures in accordance with our previous publication [1]. In summary, the healthy L. *vannamei* (shrimp) was obtained from the Hongyun farm in Zhangpu, Zhangzhou, Fujian Province. The shrimp had an average weight of 23.16 ± 2.5 g and a body length range of around 10–15 cm. The specimens were acclimated to aerated seawater maintained at a consistent temperature of 25 ± 2 °C and salinity of 28 ± 2 ppt for a duration of seven days prior to the commencement of the experiment.

#### Fungal challenge experiment and sampling

The opportunistic filamentous fungus *F. solani* was selected to conduct a challenging experiment to evaluate the four TF genes' response to fungal infection. The L. *vannamei* was distributed correspondingly into the experimental set and the control set. The inoculum from the *F. solani* pathogen was prepared with slight modifications to the method described by Goncalves, et al. [17]. Concisely, 10 µl of *F. solani* was grown in dextrose potato agar at 35 °C for seven days. We then picked the spores from the agar's surface using the saline solution, sieved them with a filter cell, and centrifuged them at 6000 rpm for 25 min. Then washed the pellet spore three times with saline, regulating the concentration with a hemocytometer. The experimental set received 25 µl of fungal inoculum spores at 1 × 10^5^ concentrations, and the control group received an equal volume of sterile saline solution. The hemocytes from six challenged and six control animals per condition were collected at 0, 12, 24, 48, 72, and 96 h (h). The plasma was immediately removed by centrifuging it at 800 g for 10 min at 4 °C, and then the cell pellet was used to extract the RNA. Similarly, the gills and hepatopancreas of both treatment and control animals were frozen at 0, 12, 24, 48, 72, and 96 h in RNA-later before the RNA extraction.

### RNA extraction, cDNA synthesis and expression analysis

The E.Z.N.A. Total RNA Kit II (Omega Bio-Tek, Norcross, GA, USA) was used to isolate the total RNA from the hemocytes, hepatopancreas, and gill tissue, adhering to the manufacturer's instructions. The Prime Script first-strand cDNA synthesis kit (Yeasen; Shanghai, China) was employed to perform reverse transcription of RNA samples from both the experimental and control groups into cDNA, adhering to the manufacturer's guidelines. The primers for all selected genes for the qRT-PCR study were generated using NCBI BLAST, which we report in Supplementary File Table S1. Roche Light Cycler 480 II equipment (Takara Dalian, China) was employed for the implementation of qRT-PCR. The overall reaction volume consisted of a 20 µl combination, comprising 9 µl of SYBR green premix, 10 µl of template cDNA, and 0.5 µl of each primer. The expression of each gene was normalized using the *ef1-α* gene of L. *vannamei*, and the relative expression of each gene was calculated using the 2^−ΔΔCT^ technique [18].

### Statistical analysis

Statistical analysis was conducted using SPSS 17.0 software, and the data were given out as the mean ± standard error (SEM). A one-way ANOVA was used to evaluate statistically significant changes, with a *P* value of less than 0.05 indicating a statistically significant difference.

## Results

### Identification of immune-related genes in L. *vannamei* genome

We identified twenty-one (21) differentially expressed genes, previously published elsewhere, in immune-related tissues of decapod crustaceans using the L. *vannamei* genome. We employed the previously reported genes as a query to conduct a blast against the L. *vannamei genome*. Table 1 provides detailed information regarding their genomic sequences, gene ID, accession, beginnings, and stop codons.

**Table 1:** List of genes expressed in hemocyte identified in the L. *vannamei* genome.

**Gene Name**	**Gene ID**	**Accession**	**Start**	**Stop**	**Chr**	**Reported species**	**Reference**
*Β-Actin*	113803353	NW-020868744.1	32560	33693	Un Scaffold	*E. carinicauda*	[36]
*Cathepsin L*	113807041	NW-020869148.1	781510	792980	Un Scaffold	*E. sinensis*	[37]
*C-type lectin A*	113825092	NW-020868325.1	327035	329216	Un Scaffold	*E. sinensis*	[37]
*C-type lectin G*	113812219	NW-020869735.1	60536	64073	Un Scaffold	*E. sinensis*	[37]
*Galectin*	113805092	NW-020868948.1	59149	63074	Un Scaffold	*M. japonicus*	[38]
*GSK-3*	113819809	NW-020870571.1	297136	297561	Un Scaffold	*E. sinensis*	[39]
*IKK*	113804545	NW-020868887.1	17297	24525	Un Scaffold	*S. paramamosain*	[40]
*Integrin*	113805722	NW-020869029.1	153535	156040	Un Scaffold	*E. sinensis*	[37]
*Kunitz-type*	113806311	NW-020869081.1	123736	126016	Un Scaffold	*P. monodon*	[41]
*LGBP*	113807222	NW-020869172.1	147145	154321	Un Scaffold	L. *vannamei*	[42]
*P38*	113815719	NW-020870106.1	322833	347715	Un Scaffold	*E. sinensis*	[37]
*Penaeidin 3a*	113808994	NW-020869378.1	53040	59884	Un Scaffold	L. *vannamei*	[37]
*Phenoloxidase-1*	113828755	NW-020872432.1	913935	917932	Un Scaffold	L. *vannamei*	[37]
*Ras-like GTPase*	113809844	NW-020869463.1	134495	141438	Un Scaffold	*M. japonicus*	[43]
*Rhodanese domain*	113830416	NW-020872637.1	884532	893128	Un Scaffold	*M. nipponensis*	[44]
*ROMO-1*	113814458	NW-020869984.1	112717	116376	Un Scaffold	L. *vannamei*	[45]
*Serpin*	113821900	NW-020870801.1	250428	252658	Un Scaffold	*E. sinensis*	[37]
*SOCs*	113827830	NW-020872191.1	306850	320703	Un Scaffold	*E. sinensis*	[46]
*SOD*	113823550	NW-020871003.1	1661	10894	Un Scaffold	*E. sinensis*	[37]
*Thymosin beta-like*	113827561	NW-020872129.1	72702	90691	Un Scaffold	*C. quadricarinatus*	[47]
*TGF-β receptor*	113808959	NW-020868425.1	301827	307800	Un Scaffold	*S. paramamosain*	[48]

### Gene structure, subcellular localization, and conserved motifs analysis

**Fig. 1:**
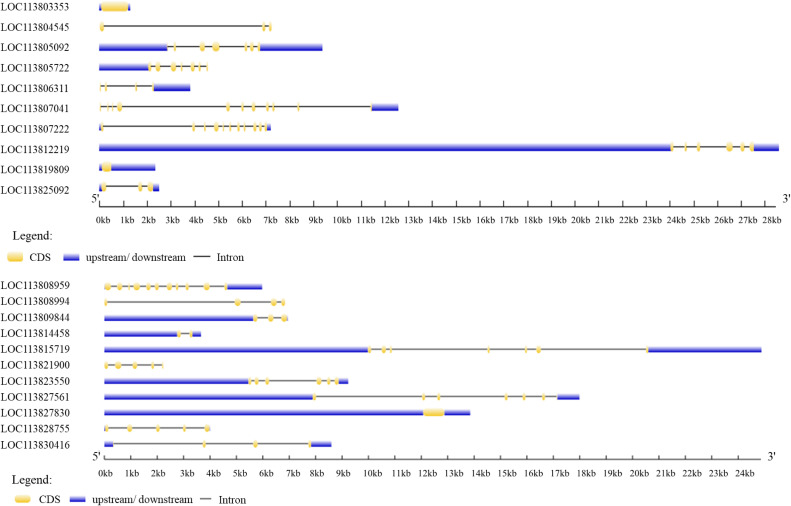
Exon/Intron distribution of the twenty-one genes (http://gsds.cbi.pku.edu.cn/).

The arrangement of exons and introns primarily reflected the influence of certain gene families [19]. Using a gene structure display server (GSDS), we aligned each gene's CDS with the genomic sequence in order to gain a deeper understanding of the structural evolution of the twenty-one genes. Various exon/intron distribution patterns were observed, as illustrated in Fig. 1. Most of the twenty-one gene is made up of introns, except for Actin-like, *SOCs*, and *GSK-3*, which have multiple repeat sequences. On the other hand, the number of introns varied from 1 to 10, showing differences among the twenty-one genes.

**Fig. 2:**
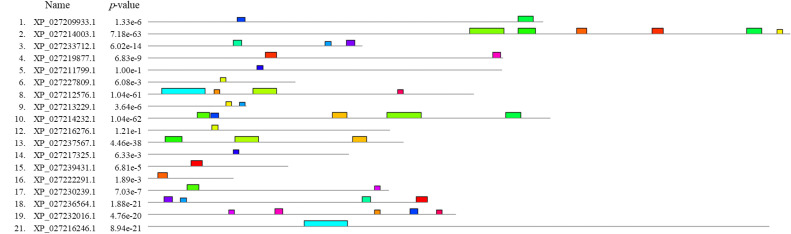
A schematic representation of conserved motifs (obtained using MEME) in twenty-one proteins. Boxes of different colors represent different motifs (http://meme-suite.org/tools/meme).

To gain a better understanding of the biological function of the twenty-one genes, we examined their various characteristics. This included factors such as molecular mass, theoretical PI, number of amino acids, and subcellular localization. Table 2 conveniently summarizes all of this information. The molecular weight of twenty-one proteins varied between 11.42 and 95.15 kDa. Out of the total of 21 proteins, 10 of them fell within the theoretical PI range of 6.00 to 9.89. As a result, the majority of proteins exhibited a consistent cellular localization, potentially accounting for the expression of those genes in crustacean hemocytes. Just like a biophysicist, it is interesting to note that the proteins in the L. *vannamei* genome share a high degree of similarity in their motif composition, as depicted in Fig. 2.

**Table 2:** Summary information of the twenty-one-protein identified in L. *vannamei* genome.

Protein ID	MW	PI	AA	Subcellular localization
XP-027209933.1	11.42	5.23	377	Cysk
XP-027214003.1	41.68	5.96	613	ER, Lyso, Golg, Mito-Pero, Plas, Extr
XP-027233712,1	40.22	5.36	205	Cyto-Nucl, Extr, Pero
XP-027219876.1	92.38	6.27	339	Cyto-Pero, ER, Golg, Lyso, Mito
XP-027211799.1	28.34	7.71	338	Cyto-Nucl, Extr, Mito, Plas
XP-027227809.1	19.16	7.61	141	Cyto-Nucl, Lyso, Extr
XP-027211239.1	83.20	9.11	189	Cyto-Nucl, Plas, Extr, Lyso
XP-027212576.1	46.35	4.74	311	ER, Lyso, Plas, Extr
XP-027213229.1	68.11	5.55	94	Extr
XP-027214232.1	74.44	4.50	384	Mito, ER, Lyso, Golg, Plas
XP-027223540.1	60.29	4.95	259	Cyto-Nucl, Cysk, Extr
XP-027216276.1	89.26	9.13	231	Cyto-Nucl, Cyto-Pero, Mito, Golg
XP-027237567.1	85.28	5.82	244	Cyto-Nucl, Extr, Pero
XP-027217324.1	16.22	6.00	192	Cyto-Nucl, Extr, Mito, Plas, Golg
XP-027239431.1	95.15	5.21	134	Cyto-Nucl, Mito, Extr
XP-027222291.1	14.87	9.89	82	Cyto-Nucl, Mito, Plas, ER
XP-027230239.1	39.26	9.44	230	Cyto-Nucl, Extr, Mito, Golg
XP-027236564.1	55.29	8.61	267	Cyto-Nucl, Cyto-Pero, Mito, Golg
XP-027232016.1	47.32	5.62	294	Cyto-Nucl, Cyto-Plas, Mito, Lyso
XP-027236256.1	66.27	5.42	242	Cyto, Extr, Nucl
XP-027216246.1	47.64	6.37	593	Plas, Nucl, Cyto-Mito

AA, amino acids; MW, molecular weight (kDa); PI, isoelectric point; Cyto, cytoplasm; ER, endoplasmic reticulum; Mito, mitochondrion; Nucl, nucleus; Lyso, lysosomes; Golg, Golgi apparatus; Plas, plasmid; Pero, peroxisome; Extr, extracellular space; Cysk, cytoskeleton.

### Identification of transcription factor family

**Fig. 3:**
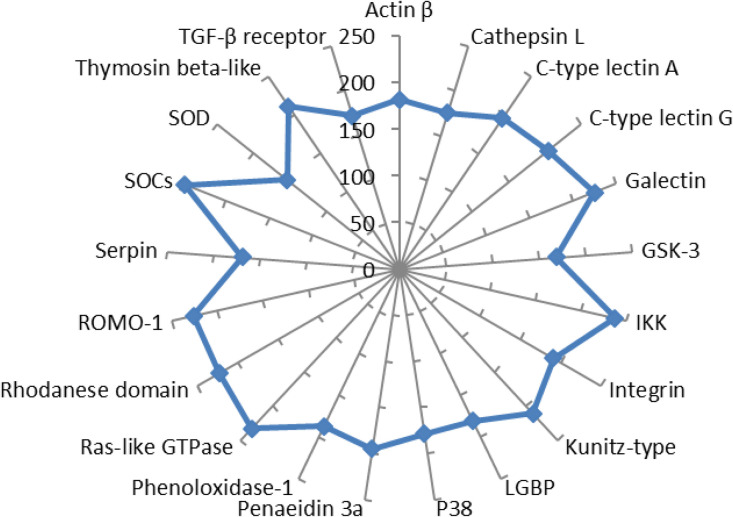
Maximum number of transcription factors for different genes.

**Fig. 4:**
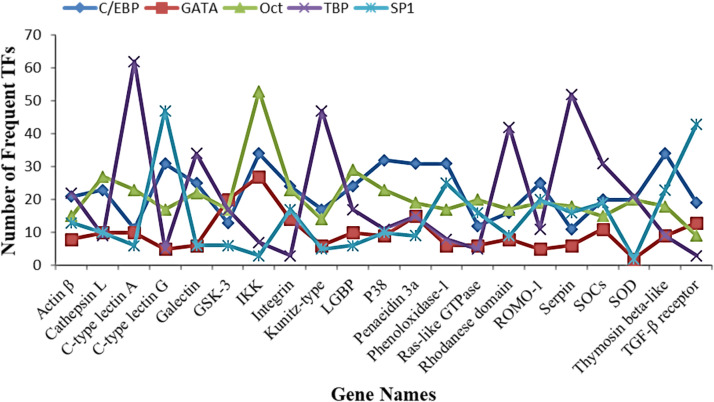
Determination of most frequent transcription factors among the analyzed genes.

To learn more about how gene expression and the transcription factors family work together in L. *vannamei*, we did a genome-wide analysis of TFs in twenty-one genes. For each gene, we downloaded a 2000-bp upstream sequence from the L. *vannamei* genome. We then used the online promoter 2.0 server to guess the transcription start sites (TSS) and the Alibaba 2.1 online program to guess the possible binding TFs. The results showed that there were 4167 TFs in 21 genes, which we report in Supplementary file Table S2. Fig. 3 shows that *SOCs* have the most TFs family members, followed by *IKK*, Ras-like *GTPase, ROMO-1*, Galectin, Kunitz-type, thymosin beta-like, and C-type lectin G, with values of 247, 233, 226, 225, 223, 211, and 204, in that order. However, five transcription factor families are the most repeated in all twenty-one genes, such as the *C/EBP, GATA, Oct, TBP*, and *SP1* transcription factor families (Fig. 4).

### Tissue expression analysis of TFs genes in Pacific white shrimp

**Fig. 5:**
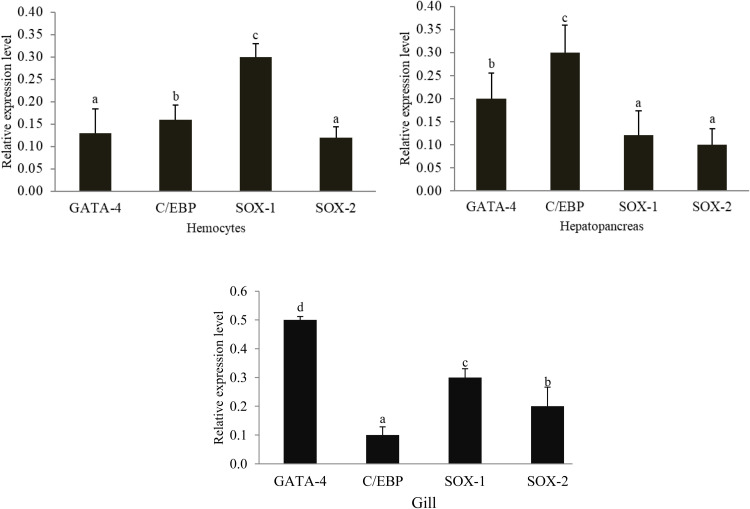
Tissue-specific expression of TFs genes in healthy tissues (hemocytes, hepatopancreas, and gill) of L. *vannamei*. The value with a different letter indicates a significant difference at *p < 0.05*.

We used quantitative real-time polymerase chain reaction (qRT-PCR) to confirm that the L. *vannamei* transcription factors (TFs) genes were expressed in the right tissues. We detected the relative expression levels in healthy tissues, including Hem, HP, and Gi. All four transcription factor genes were consistently expressed in all tested tissues, according to the results. The expression levels of the *SOX-1, C/EBPγ*, and *GATA4* genes were found to be significantly high in Hem, Hp, and Gi, respectively. Conversely, Hem and HP showed the lowest expression level of *SOX-2*. On the other hand, the Gi significantly decreased the expression level of *C/EBP* (Fig. 5).

### Expression analysis of selected TFs genes in Pacific white shrimp after fungal infection

**Fig. 6:**
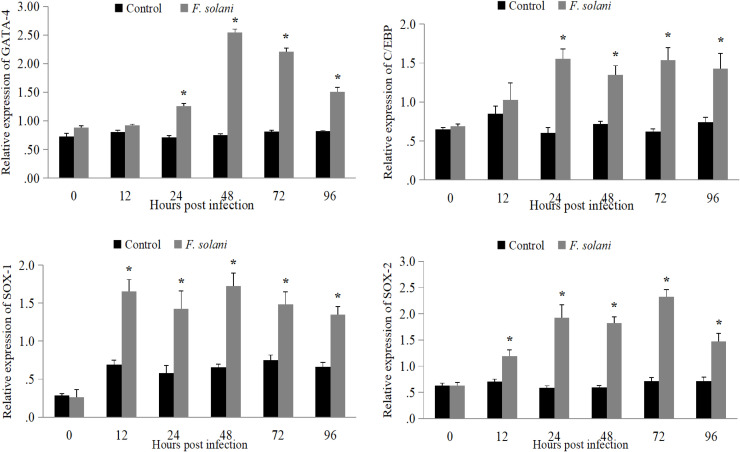
Expression profiling of *GATA4, C/EBPγ, SOX-1*, and *SOX-2* genes in L. *vannamei* hemocytes at 0, 12, 24, 48, 72, and 96 h under *F. solani* infection. The asterisk * indicates a significant difference at *p < 0.05.*

We conducted a quantitative real-time polymerase chain reaction (qRT-PCR) to measure the relative expression levels of these predicted L. *vannamei* transcription factors (TFs) genes in Hem, HP, and Gi to examine their probable participation in the response to fungal infection. The analysis of hemocytes revealed that the expression of all four TFs genes varied significantly in response to the infection as compared to the control group. After 12 h, there was a clear difference in the expression levels of the *SOX-1* and *SOX-2* genes. After 24 h, there were big changes in the expression levels of the *GATA4* and *C/EBPγ* genes (Fig. 6).

**Fig. 7:**
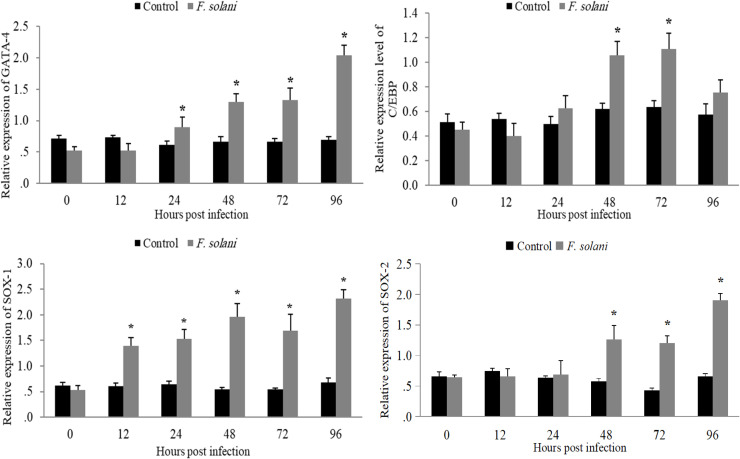
Expression profiling of *GATA4, C/EBPγ, SOX-1*, and *SOX-2* genes in L. *vannamei* hepatopancreas at 0, 12, 24, 48, 72, and 96 h under *F. solani* infection. The asterisk * indicates a significant difference at *p < 0.05.*

The four TFs genes in the hepatopancreas showed that *SOX-1* changed significantly right away, at 12 h, while the *C/EBPγ* gene changed significantly only at 48 and 72 h after infection. Moreover, treatment differentially expressed *GATA4* compared to the control group from 24 to 96 h after infection, while the *SOX-2* gene did not significantly change until 48 to 96 h after the *F. solani* challenge (Fig. 7).

**Fig. 8:**
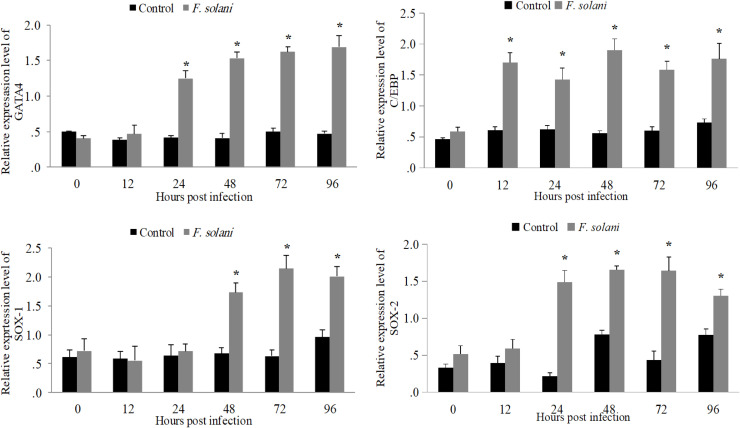
Expression profiling of *GATA4, C/EBPγ, SOX-1*, and *SOX-2* genes in L. *vannamei* gills tissue at 0, 12, 24, 48, 72, and 96 h under *F. solani* infection. The asterisk * indicates a significant difference at *p < 0.05.*

In general, all four genes exhibit a response to fungal infection by altering their transcription patterns over time in comparison to the control group. During the initial period (0–24 h post infection), the levels of *SOX-1* and *SOX-2* in infected prawns are consistent with those in the control group. A significant increase is observed between 24- and 48-hours post infection, with levels reaching their peak at 72- and 96-hours post infection, at which point the differences are statistically significant. *GATA4* expression also rises significantly from 24 h post infection, continuing to increase at 48-, 72-, and 96-hours post infection, demonstrating substantial differences from the control group. *C/EBPγ* displays a similar trend, characterized by minor fluctuations initially, followed by a pronounced increase in expression beginning at 24 h post infection, peaking later (72–96 h post infection), with significant differences noted compared to the control group. Conversely, the control group maintains a relatively stable expression level throughout, exhibiting only minor changes without any significant variations. The results indicate that all four genes respond to *F. solani* infection, displaying a general trend of increased expression in the latter stages of the infection. This implies that they may contribute to the immunological response of the host's gill tissue (Fig. 8).

## Discussion

In crustaceans, hemocytes, gills, and the hepatopancreas are recognized as the primary immune organs responsible for pathogen identification, phagocytosis, and the synthesis of immune-related chemicals [20]. Numerous studies have shown that these tissues play a crucial role in host defense and harbor a diverse array of immune-related genes [21,22]. However, the regulatory mechanisms that influence immune gene expression, particularly the role of transcription factors (TFs), remain insufficiently understood in prawn species such as *Litopenaeus vannamei*. As the disease burden in aquaculture systems rises, elucidating transcriptional regulation is crucial for comprehending host-pathogen interactions at the molecular level [23].

While viral pathogens like white spot syndrome virus (*WSSV*) and bacterial pathogens like *Vibrio* spp. have been studied a lot, fungal pathogens like *Fusarium solani* are becoming more important but are still not well-understood threats in prawn aquaculture [24,25]. Environmental stressors, elevated stocking densities, and suboptimal water quality may promote fungal proliferation; however, the host transcriptional responses to these infections remain inadequately defined. So, this study gives us new information about how TFs control the immune response in shrimp when they are exposed to fungi. This helps us understand shrimp immunity better.

The genome-wide identification of 21 TF-related genes in L. *vannamei* demonstrated significant conservation in sequence characteristics and motif composition. This kind of conservation fits with what we already know: that TFs that are important for things like development, immune regulation, apoptosis, and stress responses are evolutionarily preserved across taxa [26]. Gene structure analysis showed that there were big effects on regulation. We found that most of the genes had more than one intron. This supports the idea that genes with a lot of introns are often linked to complex and tightly controlled expression patterns [27]. On the other hand, the fact that there are genes without introns, such as Actin-like, *GSK3*, and *SOCs*, shows that it is beneficial to be able to quickly respond to stress by changing how genes are transcribed. Researchers have shown in the past that genes without introns can be transcribed and processed more quickly, avoiding delays caused by splicing. This may be especially helpful when someone is very stressed or sick [28]. Genes possessing introns may enhance the stability of mRNA and offer regulators increased flexibility, thus enabling more precise control over gene expression [29]. This dual structure of genes could potentially strengthen the immune system of L. *vannamei*, allowing for a rapid and coordinated response.

Promoter analysis showed that the *C/EBP, GATA, Oct, TBP*, and *SP1* TF families had a lot of predicted binding sites, which suggests that they might be involved in controlling the genes that were found. But you should be careful when you look at these results. TFs like *TBP* and *SP1* are general transcriptional regulators that play a role in basal transcription. Their high frequency may be due to their involvement in general promoter architecture rather than specific immune regulation [30,31]. On the other hand, TF families like *C/EBP* and *GATA* are more often linked to regulating genes that are specific to certain tissues or the immune system [32,33], which makes them better candidates for functional validation. Taking all of this into account, we chose *GATA4, C/EBPγ, SOX-1*, and *SOX-2* for experimental testing. *GATA* and *C/EBP* families are well-known for controlling immune and developmental processes. The addition of *SOX* transcription factors shows that they are becoming more important in cell differentiation, tissue homeostasis, and stress responses [34]. *SOX* TFs weren't one of the most common predicted families, but their role in transcriptional reprogramming suggests that they might help the host adapt to an infection.

Expression profiling shows that these TF genes are always active in important immune-related organs like hemocytes, hepatopancreas, and gills. This suggests that they are important for keeping the body's normal balance. More importantly, their different levels of expression after *F. solani* infection show how responsive they are to fungal challenges. These TFs may act as regulatory centers that control the expression of immune genes downstream, as seen in the patterns of expression that change over time and in different tissues. In the context of bacterial and viral infections, analogous inducible expression patterns of transcription factors have been observed in prawns, thereby affirming their role as crucial mediators of host defense [35]. Nonetheless, this study has specific limitations. Expression profiling and in silico analysis were the primary methodologies employed to ascertain the functional roles of the identified transcription factors. To confirm their regulatory functions, TF–target gene interactions must be directly validated through methodologies such as chromatin immunoprecipitation or gene knockdown. Additional research contrasting bacterial, viral, and fungal challenges would assist in elucidating whether these transcription factors function as general stress-responsive entities or exhibit pathogen-specific roles. This study concludes with a comprehensive analysis of TF families in L. *vannamei*, highlighting their potential roles in regulating immune responses during fungal infections. Our results, which combine bioinformatics and expression analysis, help us understand how transcriptional regulation works in prawns. They could also help with selective breeding and disease control in aquaculture in the future.

## Conclusion

In this study, we identified an exhaustive set of 4167 TF families and determined that five TFs (C/EBP, GATA, Octamer, TBP, and Sp1) were the most frequently occurring across the twenty-one genes. We hypothesized that these five most frequently occurring transcription factors could significantly influence the expression of those genes. Structure and function studies of the twenty-one genes show that the Actin-β-like, GSK3, and SOC genes do not have any introns. Conversely, the remaining genes contain numerous introns and conserved motifs. Our genome-wide systemic characterization has provided us with a clue to comprehend the molecular function of these genes and TFs, which will serve as the foundation for our future research. The expression patterns of the four TF genes were found to be diverse in three distinct normal tissues of L. *vannamei*, as indicated by expression analysis. It is intriguing that the expression levels of *SOX-1* genes in L. *vannamei* hemocytes, hepatopancreas, and gills were substantially elevated in response to *F. solani* infection.

## Funding

This research was funded by Seed Industry Innovation and Industrialization Engineering Marine and Fishery Project of Fujian Province (2017FJSCZY02).

## Informed consent

Not applicable.

## Ethics approval

The study design and animal experiments were meticulously crafted to align with the guidelines set forth by the Animal Care and Use Committee of Fujian Agriculture and Forestry University.

## CRediT authorship contribution statement

**Yusuf Jibril Habib:** Writing – review & editing, Writing – original draft, Visualization, Validation, Software, Resources, Methodology, Investigation, Data curation, Conceptualization. **Akram Ismael Shehata:** Writing – review & editing, Visualization, Validation, Methodology, Investigation, Formal analysis. **Chengjie Yao:** Writing – review & editing, Methodology, Data curation. **Haifu Wan:** Writing – review & editing, Methodology, Data curation. **Jiaming Lin:** Writing – review & editing, Methodology. **Hui Ge:** Writing – review & editing, Methodology, Data curation. **Mohammed F. El Basuini:** Writing – review & editing, Investigation, Data curation. **Mayada Alhoshy:** Writing – review & editing, Visualization, Validation, Investigation, Data curation. **Yilei Wang:** Writing – review & editing, Supervision, Resources, Conceptualization. **Ziping Zhang:** Writing – review & editing, Visualization, Validation, Supervision, Resources, Investigation, Conceptualization.

## Declaration of competing interest

The authors declare no conflict of interests.

## Data Availability

Data will be made available on request.

## Appendix Supplementary materials

Image, application 1
