# Supplementary material for: The predicted transcription factors of the immune-related genes in Pacific white shrimp (Litopenaeus vannamei) respond to Fusarium solani infection
Source: Comp Immunol Rep. 2026 May 3;10:200288. doi: 10.1016/j.cirep.2026.200288 (PMC13187582; doi:10.1016/j.cirep.2026.200288)
Supplement: Supplementary file 1 [file mmc1.docx]

**Table S1:** Primers of transcription factor genes used in this study

| **Gene Name** | **Gene ID** | **qPCR Primer** |
| --- | --- | --- |
| *GATA-4* | 113820974 | **F** AGAATGTGTGAATTGCGGCG |
|  |  | **R** AGGTTTGGAAGGAGAAGCCG |
| *C/EBP gamma* | 113814445 | **F** TGGAAGGACTGAGTGGGGAA |
|  |  | **R** GACGCCTGCGATACTCATCA |
| *SOX-1* | 113829620 | **F** ACCCTTATTGTCGCGTGTGT |
|  |  | **R** CAAAGCGCGATACTTGTCCG |
| *SOX-2* | 113828508 | **F** GTCGGTTCCGTGGGTTCTTC |
|  |  | **R** TTCATCACGCTCGCTGGTGT |
| *Ef1α* | 113820946 | **F** ACCATCATCGATGCCCCAGG |
|  |  | **R** CAGCTTCGAACTCGCCGGTA |

**Table** **S2**: Detailed information of the predicted transcription factors of the twenty-one immune-related genes

| **Gene names** | **Species Name** | **Gene ID** | **Chr** | **No. of TF** | **Name of Transcriptional factors** |
| --- | --- | --- | --- | --- | --- |
| Actin-like | *L. vannamei* | 113803355 | 20868744.1 | 182 | C/EBPbeta, HNF-1C, NF-1, COUP, REV-ErbA, ER, Oct-1, NF-1, NF-1, GATA-1, GATA-1, Ftz, TBP, Oct-1, PEA3, GR, Hb, C/EBPalpha, GATA-1, NF-ATc3, HNF-1C, Oct-1HNF-3B, PR, Sp1, NF-kappa, Ftz, GR, GATA-1, Oct-1, TBP, GR, GCN4, Hb, MEB-1, SRF, MEB-1, C/EBPalp, GCN4, C/EBPalp, TBP, Antp, GATA-1, C/EBPalp, Oct-1, C/EBPalpHNF-1, TBP, RAP1, Oct-1, Oct-2.1, C/EBPalp, ICSBP, AP-1, AP-1, AP-1, AP-1, MCM1, SRF, C/EBP, Oct-1A, NF-kappaB, CBP100, Oct-1, AP-1, C/EBPalp, NF-1, Sp1, IRF-1NF-kappa, RAP1, RAP1, RAP1, Oct-1, GATA-1, TBP, Sox-2, WT1_I_-K, WT1_I, WT1-del2, WT1_I-de, WT1_I_-K, Sox-2, WT1_I, WT1-del2, WT1_I-de, Sox-2, SRF, RAP1TBP, NF-1, TBP, TBP, Pit-1a, TBP, TBP, TBP, Pit-1a, TBP, Pit-1a, TBP, Ftz, E2, c-Jun, CREB, C/EBPalp, CPE_bind, AP-1, CRE-BP1, GR, USF, C/EBPalp, TBP, TBP, TBP, Pit-1a,Oct-1, Ftz, TBP, TBP, TBP, TBP, Sp1, Sp1, C/EBPalp, Oct-1, Sp1, WT1, NRF-1, NRF-1, ARP-1, Sp1, C/EBPbeta, C/EBPbeta, Oct-1, HNF-3, TBP, C/EBPbeta, Hb, GATA-1, TBP,GATA-1, HNF-1C, Odd, Sp1, C/EBPalp, NF-kappaB, p40x, AP-1, NF-1, Oct-2.1, C/EBPalp, Dfd, HNF-1, CPE_bind, AP-1, MRF4, RXR-beta, CRE-BP1, MIG1, Sp1, Oct-1, USFGR, CPE_bind, CRE-BP1, CREB, C/EBPalp, AP-1, c-Jun, C/EBPalp, AP-2alphaA, Sp1, C/EBPdel, SRF, C/EBPdel, AP-2alph, Sp1, Sp1, Sp1, TBP, Sp1 |
| Cathepsin L | *L. vannamei* | 113807041 | 20869148.1 | 175 | TBP, GATA-1, Oct-1, Zen-1, HNF-3, C/EBPalp, Oct-1, NF-1, RAP1, GR, PU.1, IRF-1, Hb, Pit-1, Oct-2.1, Pit-1a, TBP, C/EBPalp, Oct-11, TBP, C/EBPalp, Oct-11, TBP, C/EBPalpErg-1, GCN4, AP-1, AP-1, c-Jun, ER, Oct-1A, Oct-1, C/EBPalp, TBP, GR, C/EBPalpha, TBP, GAL4, C/EBPalp, Oct-1, Ftz, GATA-1, Oct-1, TBP, RSRFC4,TBP, Hb, GLO, Sp1MEB-1, Pit-1a, Oct-11, Oct-11, GATA-1, GATA-1, Oct-1A, GR, C/EBPalp, Ftz, CP1, c-Fos, GCN4, C/EBPalp, C/EBPalp, ICSBP, SRF, ISGF-3, Sp1, Oct-11, C/EBPalp, C/EBPalpCOUP, GR, PR, Hb, C/EBPalp, HNF-3, MEB-1, C/EBPalp, Dfd, HNF-1C, Id3, NF-ATc3, Oct-6, Oct-1,GATA-1, RAP1, SRF, C/EBPalp, C/EBP, ICSBP, HNF-1C, NF-1, Elk-1C/EBPalp, Oct-2.1, NF-A, GATA-1, PEA3, GR, C/EBPalp, GCN4, HNF-3, Hb, Egr-1, ETF, Sp1, WT1, Ttx, RAR-al, RXR-b, T3R-alph, etaph, ER, CPE_bind, NF-1, GATA-1, Sp1 PR, GR, Oct-1, GR, Pit-1a, GATA-1, TBP, Oct-1, Pit-1a, Oct-1, Oct-1, Oct-1, Elk-1, C/EBPalp, MEB-1, GLO, Oct-1, MEB-1, GATA-1, NF-1, MCM1, GR, NF-1, NF-1, HSTFNF-kappaB, C/EBPalpha, C/EBPalp, Oct-1, GATA-1, Oct-1, SRF, Oct-1, C/EBP, Sp1, Sp1, ER, Oct-1, Sp1, MyoD, Sp1, AP-2alph, USF, Sp1, C/EBPbeta, C/EBPalp, Oct-1, RAP1MEF-2, NF-1, E1, MyoD, PEA3, USF, AP-1, Sp1, c-Jun, ER |
| C-type lectin A | *L. vannamei* | 113825092 | 20868325.1 | 196 | Ftz, NF-EM5, GATA-1, Oct-1, AP-1, MATalpha1, HNF-1, Sp1, CRE-BP1, CREB, C/EBPalp, CPE_bind, ATF, HNF-1C, MATalpha2, C/EBPalp, SGF-3, Oct-1A, Oct-1, Oct-11,GATA-1, Ubx, C/EBPbeta, HNF-1C, Oct-1A, HNF-1, Oct-1, C/EBPalp, Pit-1a, Vmw65, C/EBPalp, GATA-1, Ubx, Zen-1, GATA-1, Oct-1, Oct-1, C/EBP, Antp, Ubx, Oct-11,GATA-1, Oct-1, C/EBPalp, GATA-1, C/EBPdelta, GATA-1, NF-kappaB, NF-kappaB, Oct-11, MEB-1, C/EBPalp, RAP1, RAP1, RAP1, Sp1, RAP1, RAP1, Sp1, RAP1,RAP1,Oct-1, GR, Sp1, C/EBPalp, Hlf, Oct-1, Oct-1, GR, Oct-1, Sp1, GATA-1, PTF1-beta, Dl, Oct-1, WT1_I_-K, Sox-2, W T1_I, WT1-del2, WT1_I-de, Sp1, RAP1, Sox-2, USF, Oct-1,TBP, TBP, Ftz, TBP, TBP, TBP, TBP, TBP, TBP, TBP, TBP, TBP, TBP, TBP, TBP, Ftz, TBP, TBP, TBP, TBP, TBP, Pit-1a, Oct-1, TBP, C/EBPalp, SRF, TBP, Pit-1a, SRF, TBP,Pit-1a, TBP, TBP, TBP, TBP, Oct-1, TBP, TBP, TBP, TBP, TBP, TBP, TBP, TBP, SRF, RAP1, RAP1, RAP1, RAP1, TBP, TBP, Pit-1a, TBP, TBP, TBP, TBP, TBP, TBP, TBP, TBP,Pit-1a, TBP, Oct-1, TBP, TBP, TBP, TBP, Ftz, TBP, TBP, Pit-1a, TBP, WT1_I_-K, Sox-2, WT1_I, WT1-del2, WT1_I-de, WT1_I_-K, Sox-2, WT1_I, WT1-del2, WT1_I-de, SRF,TBP, TBP, TBP, TBP, TBP, TBP, USF, TBP, GATA-1, Oct-1, SRF, TBP, Pit-1a, Oct-1, TBP, Sp1, GATA-1, Odd, HNF-3, SRF, TBP, Pit-1a, HNF-3, TBP, Pit-1a, Oct-1, SRF, TBP |
| C-type lectin G | *L. vannamei* | 113812219 | 20869735.1 | 204 | TBP, HNF-3, Oct-1, Pit-1a, Oct-1, C/EBPalp, HNF -1, C/EBPalp, Ftz, Oct-1, C/EBPalp, Hb, HNF-3B, C/EBPalp, Id3, HNF-1, NF-ATc3, NF-1, Oct-1, LyF-1, NF-1, NF-1, C/EBPalp,HNF-1, C/EBPdelta, ICSBP, C/EBPalp, Hb, ICSBP, Olf-1, NF-kappa, GATA-1, GATA-1, TBP, SRF, TBP, C/EBPalp, Oct-1, C/EBPalp, GR, Erg-1, NF-1, RSRFC9, IRF-1, MEB-1, Pit-1a, GR, Pit-1a, Oct-1, C/EBPalp, C/EBPgam, Hb, Pit-1a, Oct-1, C/EBPalp, HNF-3, Pit-1a, Oct-1, C/EBPbeta, GATA-1, Elk-1, C/EBP, IRF-1, Sp1, GR, C/EBPbeta, AP-1, AP-1, Ubx, Hb, C/EBPalp, Hb, p40x, Oct-1, C/EBPbeta, HOXA4, Oct-1, Pit-1, C/EBPalp, NF-1, HNF-3, YY1, C/EBPalp, NF-1, TBP, Oct-1, Pit-1a, C/EBP, AP-1, Oct-1, HNF-1, NF-1, C/EBPalp, DBP, Oct-1, RSRFC4, GLO, C/EBPalp, HNF-1C, HNF-3, C/EBPalpha, Oct-1, Ftz, Oct-1, HNF-1, C/EBPalp, Oct-11, GATA-1, C/EBPalp, Ttx, NF-1, AP-1, GATA-1, Oct-1, HOXA4, Odd, Zen-1, Sp1, Pit-1a, C/EBPalp, Hb, C/EBPalp, SRF, C/EBPalp, Hb, C/EBPbeta, ER, ETF, Sp1, AP-2, NF-1, TEC1, Sp1, GR, TBP, PU.1, Sp1, Sp1, ICSBP, ISGF-3, Sp1, TBP, GCN4, C/EBPalpha, c-Fos, AP-1, ETF, Sp1, AP-2, Sp1, Sp1, TBP, C/EBPdel, GCN4, AP-1, AP-1, Sp1, Sp1, Krox-20, AP-2, Sp1, Sp1, ETF, Sp1, Sp1, TBP, Elf-1, C/EBPalp, AP-1, Sp1, ETF, Sp1, MRF4, Sp1, Sp1, Sp1, Sp1, Sp1, Sp1, Sp1, Sp1, Sp1, Sp1, Sp1, PU.1, Sp1, Sp1, Sp1, Sp1, Sp1, Sp1, Sp1, Sp1, Sp1, Sp1, Sp1, Sp1, Sp1, Sp1, Sp1, Sp1, Sp1, Sp1, Ttx. |
| Galectin | *L. vannamei* | 113805092 | 20868948.1 | 225 | Oct-2.1, c-Jun, c-Fos, GCN4, AP-1, HNF-1, Oct-2.1, C/EBPalp, RSRFC4, TBP, C/EBPalp, C/EBPdel, Ftz, NF-1, NF-1, C/EBPalpha, Hb, HNF-3, NF-ATc3, Ftz, HNF-1, Pit-1a, PU.1, TBP, GAL4, ISGF-3, ICSBP, C/EBPalp, Zen-1, Oct-1, c-Ets-1, CeMyoD, GATA-1, CP1, C/EBPalp, Sp1, NF-kappaB, NF-1, GATA-1, Sp1, Sp1, GR, NF-1, C/EBPbeta, NF-1, CPE_bind, CBP100, CRE-BP1, IRF-1, C/EBP, Hb, C/EBPeps, TSF3, MPBF, Oct-1, Pit-1a, C/EBPalp, C/EBPbeta, TBP, SRF, C/EBPalp, Oct-1, NF-1, C/EBPalp, Pit-1a, Oct-1A, MEB-1, TBP, Oct-1, NF-kappaB, SRF, C/EBPalp, C/EBPgam, Oct-1, C/EBPalp, Vmw65, TEC1, C/EBPalp, HNF-1, Oct-1A, C/EBPdel, MyoD, ER, TBP, C/EBPdel, HSTF, Sp1, NF-kappaB, GATA-1, Dl, SRF, C/EBPalp, Oct-11, Oct-1, C/EBPbeta, Glass, NF-kappaB, WT1_I_-K, WT1_I_-K, Sox-2, WT1_I, WT1-del2, WT1_I-de, WT1_I_-K, Sox-2, WT1_I, WT1-del2, WT1_I-de, WT1_I_-K, Sox-2, WT1_I, WT1-del2, WT1_I-de, WT1_I_-K, Sox-2, WT1_I, WT1-del2, WT1_I-de, WT1_I_-K, Sox-2, WT1_I, WT1-del2, WT1_I-de, WT1_I_-K, Sox-2, WT1_I, WT1-del2, WT1_I-de, Sox-2, SRF, TBP, TBP, Pit-1a, TBP, TBP, Pit-1a, Ftz, TBP, Pit-1a, TBP, TBP, TBP, TBP, Oct-1, SRF, TBP, Ftz, TBP, TBP, SRF, RAP1, RAP1, RAP1, RAP1, C/EBPbeta, Oct-1, RAP1, Oct-1, TBP, SRF, Pit-1a, TBP, Oct-1, NF-A, SRF, TBP, TBP, Ftz, Pit-1a, TBP, TBP, Oct-1, TBP, TBP, Oct-1A, TBP, TBP, TBP, TBP, SRF, RAP1, Sp1, RAP1, RAP1, RAP1, RAP1, Oct-1, TBP, TBP, TBP, TBP, TEF, Pit-1a, TBP, DBP, C/EBPalp, Oct-1, TBP, MEB-1, Antp, Oct-1, Pit-1a, RAP1, Oct-1, GATA-1, GATA-1, Erg-1, AP-1, YY1, C/EBPalp, GATA-1, Oct-1, SOX-9, NF-A, HNF-3, Hb, MCM1, SRF,YY1, Emc, Id3, NF-ATc3, NF-1, C/EBPalp, Sp1 |
| GSK-3 | *L. vannamei* | 113826232 | 20871628.1 | 169 | GR, myogenin, T3R, C/EBP, Oct-1, GATA-1, Hb, c-Jun, c-Fos, Pap1+, AP-1, AP-1, GATA-1, GATA-1, Oct-1, PEA3, Pit-1a, C/EBPbeta, C/EBPalp, Oct-1, GATA-1, Ftz, Sp1, YY1, Oct-1, GR, GATA-1, GATA-1, PHO2, GATA-1, GR, GATA-1, PHO2, GATA-1, GATA-1, PHO2, GATA-1, CRE-BP1, GATA-1, C/EBPalp, GATA-1, GATA-1, GATA-1, TBP, TBP, TBP, Pit-1a, TBP, Oct-1, C/EBPbeta, Oct-11, TBP, TBP, Pit-1a, TBP, Oct-1, USF, SRF,Pit-1a, TBP, GATA-1, TBP, MEB-1, TBP, C/EBPalp, TBP, TBP, Pit-1a, Oct-1, TBP, TBP, TBP, TBP, SRF, Sp1, Sp1, RAP1, Oct-1, RAP1, RAP1, C/EBPgam, RAP1, RAP1, NRF-1, NRF-1, RAP1, Sp1, RAP1, Sp1, RAP1, RAP1, RAP1, RAP1, RAP1, Sp1, GLO, Ubx, Oct-1, Oct-1, Ubx, GATA-1, GCN4, HNF-3, Hb, LyF-1, C/EBPalpha, GATA-1, Oct-1, ICSBP, Oct-1, GATA-1, Ubx, NF-1, C/EBPdel, Zen-1, C/EBPalp, GLO, MEB-1, Oct-11, NF-1, NF-ATc3, NF-1, SRF, GATA-1, Oct-1, Eve, NF-1, Sp1, C/EBPalp, C/EBPalp, Id3, HNF-1, AFP1, Pit-1a, Oct-6, TBP, C/EBPalp, Hb, Oct-1, MEB-1, GLO, HNF-1, RAP1, Hb, C/EBPalpha |
| IKK | *L. vannamei* | 113804545 | 20868887.1 | 237 | GATA-1, C/EBPdel, p40x, GATA-1, Ubx, C/EBPalp, Id3, HNF-1C, NF-ATc3, Ftz, Antp, GATA-1, Oct-1, Ftz, TBP, Dfd, C/EBPalp, Ftz, Ftz, Vmw65, Oct-1, C/EBPalpha, Antp, GLO, Oct-1, Ftz, C/EBPalp, Oct-1A, Oct-1, GR, Id3, HNF-1, NF-ATc3, AFP1, Ftz, C/EBPalpha, Antp, GATA-1, Vmw65, Oct-11, Pit-1b, TBP, SGF-3, Oct-1A, GATA-1, Oct-1, Erg-1, GATA-1, Ubx, C/EBPalp, Oct-1, MEB-1, GATA-1, Antp, Ubx, Oct-1, GATA-1, Antp, Oct-1, Antp, Oct-1, C/EBPalp, Oct-1, C/EBPalp, GATA-1, Ubx, Oct-1, SGF-3, Sp1, ICSBP, Hb, RSRFC4, C/EBPalp, Oct-2.1, C/EBPbeta, Hb, NF-1, NF-kappaB, NF-kappaB, Dfd, NF-kappaB, Oct-1, C/EBPdel, HNF-1, Fra-2, Ftz, Antp, NF-1, C/EBPdel, Antp, Oct-1, GATA-1, Oct-11, GATA-1, Ubx, Oct-1, Ubx, Oct-11, TBP, Antp, Oct-1A, GATA-1, Ubx, Oct-1, TBP, C/EBPalp, Antp, Oct-1, GATA-1, CRE-CBPbind, CPE_BP1, Antp, Pit-1a, GCN4, Ubx, Oct-1, GATA-1, Oct-11, C/EBPalp, Ubx, Oct-1, GATA-1, Ubx, Oct-1, Ftz, Oct-1, Antp, Oct-1, GATA-1, Oct-11, GATA-1, Ubx, Oct-1, GATA-1, Ubx, Oct-1, Antp, GATA-1, Ubx, Oct-1, GATA-1, Oct-1, C/EBPalp, Pit-1a, Antp, MEB-1, Ftz, Ubx, GATA-1, Oct-1, Oct-1A, Oct-6, SOX-9, Ubx, GATA-1, Oct-1, TBP, Oct-11, Ubx, Oct-1, C/EBPalp, Oct-1, Zen-1, C/EBP, GLO, Oct-1, Ftz, SRF, Oct-1A, C/EBPdel, Antp, Oct-1, p40x, Ftz, MEB-1, GLO, Ftz, GATA-1, Ubx, GATA-1, Antp, Ubx, Oct-1, GATA-1, Oct-1, C/EBPalp, SOX-9, HNF-1C, GATA-1, C/EBPalp, TBP, C/EBPalp, Oct-11, Dfd, Oct-1, GATA-1, C/EBPalp, Hb, Oct-1, Oct-6, p40x, C/EBPalp, Hb, NF-1, C/EBPalp, NF-kappaB, Pit-1a, C/EBPalp, Sp1, GR, C/EBPalp, REV-ErbA, SRF, CFF, Sp1, HNF-3, Oct-1, /EBPalp, C/EBPalp, PR,NF-1, AP-1, C/EBPalp, IRF-1, TBP, Oct-1, C/EBPalp, C/EBPalpha, Sp1  HNF-1, C/EBPdel, Oct-1, C/EBPalp, RSRFC4, Hb, AP-1 |
| Integrin | *L. vannamei* | 113805722 | 20869029.1 | 191 | Ftz, HNF-1C, C/EBPbeta, Oct-1, C/EBPalp, Oct-1, ICSBP, C/EBPalpha, Hb, C/EBPalp, NF-1, NF-1, C/EBPalp, ISGF-3, Elk-1, GATA-1, Oct-1, ICSBP, MATalpha2, Oct-1, Oct-1, C/EBPalp, C/EBPalp, Oct-1, GATA-1, NF-1, GBF2, YY1, ICSBP, ER, RAR-alph, c-Jun, c-Fos, Oct-1, Sp1, Hb, GAL4, NF-1, GATA-1, NF-kappaB, Oct-2.1, Hb, C/EBPdelta, Oct-1, Sp1, Krox-20, Sp1, C/EBPbeta, Adf-1, Sp1, Sp1, Sp1, NF-1, AP-1, Ftz, C/EBPalp, AP-2alph, CPC1, Oct-1, NF-1, GATA-1, NF-1, Oct-1, Oct-1A, CP1, NF-1, ENKTF-1, USF, C/EBPalp, c-Jun, CPE_bind, CRE-Bind, ATFp1, MEB-1, GLO, GATA-1, alpha-CP1, GATA-1, TEC1, Pit-1a, C/EBPbeta, C/EBPgam, Oct-1, NF-EM5, C/EBPbeta, EmBP-1, HBP-1b, USF, GBF1, C/EBPalp, YY1, YY1, Sp1, WT1, Sp1, RAR-alph, RXR-alpha, RAR-beta, ER, CPE_bind, Oct-1, ISGF-3, ICSBP, C/EBPalp, HNF-3, TBP, C/EBPalp, GR, PR, YY1, RAP1, Sp1, c-Jun, CRE-BPa, RXR-beta, USF, NF-1, Sp1, E1, ER, GATA-1, C/EBPalp, GATA-1, Erg-1, TBP, GATA-1, Oct-1, Oct-1, MyoD, GATA-1, HNF-1, Sp1, Sp1, RAP1, Oct-1, CREMdelt, CRE-BP1, Adf-1, AP-1, Sp1, Sp1, Sp1, NF-1, USF, ER, Oct-1, HNF-3, Oct-1, C/EBPalp, Hb, GR, TBP, GATA-1, Dfd, HNF-3B, Oct-1, C/EBPalp, TEC1, GATA-1, C/EBPalp, Oct-2.1, Odd, c-Rel, NF-kappaB, GATA-1, COUP, T3R-beta1, T3R-alph, ATF-3del, Ttx, ER, RAR-alph, RXR-beta, RAR-beta, TEC1, Oct-11, Id3, NF-ATc3, Sp1, NF-1, CRE-BP1, Oct-1, C/EBP, RAP1, ER, C/EBPalp, ARP-1, Sp1, GATA-1, C/EBPalp, SGF-1 |
| Kunitz-type SPI | *L. vannamei* | 113806311 | 020869081.1 | 211 | SRF, MEB-1, Hb, HNF-3B, Oct-1, NF-kappaB, GATA-1, ICSBP, ISGF-3, Oct-2.1, C/EBPalp, SRF, MEB-1, HNF-3B, Sp1, Pit-1a, ICSBP, HNF-3, C/EBPalp, ICSBP, Dl, PU.1, TEC1,C/EBPalp, C/EBPalp, MEB-1, C/EBPdel, C/EBPalp, Hb, GATA-1, TBP, Ftz, TBP, TBP, TBP, TBP, TBP, Ftz, TBP, TBP, TBP, Ftz, TBP, TBP, TBP, TBP, TBP, TBP, TBP, TBP, Ftz, TBP, TBP, TBP, TBP, TBP, TBP, TBP, Vmw65, C/EBPalp, TBP, Oct-1, TBP, Oct-11, Antp, TBP, Oct-1, TBP, Pit-1a, Oct-1, Ftz, TBP, TBP, TBP, Oct-1, WT1_I_-K, Sox-2, WT1_I, WT1-del2, WT1_I-de, WT1_I_-KTS, Sox-2, WT1_I, WT1-del2, WT1_I-del2, WT1_I_-K, Sox-2, WT1_I, WT1-del2, WT1_I-de, WT1_I_-K, Sox-2, WT1_I, WT1-del2, WT1_I-de, WT1_I_-K, Sox-2, WT1_I, WT1-del2, WT1_I-de, WT1_I_-K, Sox-2, WT1_I, WT1-del2, WT1_I-de, RAP1, WT1_I_-K, Sox-2, WT1_I, WT1-del2, WT1_I-de, TBP, TBP, TBP, TBP, TBP, MEB-1, GATA-1, C/EBPalp, C/EBPalpha, TBP, HNF-3B, Oct-1, Antp, YY1, NF-muE1, C/EBPalp, Id3, HNF-1C, NF-ATc3, NF-1, GR, C/EBPalp, Hb, HNF-3, GATA-1, C/EBPdel, REB1, TBP, Oct-1, C/EBPalp, HNF-1, ER, C/EBPbeta, AP-1, MyoD, USF, Oct-1, Hb, NF-EM5, GATA-1, NF-1, Oct-1, GR, C/EBPalp, Sp1, E1, MyoD, C/EBPalp, AP-1, CRE-BP1, Sp1, LyF-1, Sp1, C/EBPalp, Oct-1, Sp1, GATA-1, Oct-1, TBP, Pit-1a, Ftz, TBP, TBP, TBP, TBP, TBP, Pit-1a, TBP, TBP, TBP, Oct-1, SRF, TBP, TEF, HNF-3, WT1_I_-K, Sox-2, WT1_I, WT1-del2, WT1_I-de, WT1_I_-KTS, Sox-2, WT1_I, WT1-del, WT1_I-del2, WT1_I_-K, Sox-2, WT1_I, WT1-del2, WT1_I-de, WT1_I_-K, Sox-2, WT1_I, WT1-del, WT1_I-de, WT1_I_-K, Sox-2, WT1_I, WT1-del2, WT1_I-de |
| LGBP | *L. vannamei* | 113807222 | 20869172.1 | 181 | C/EBPalp, GR, C/EBPalp, Oct-1, C/EBPbeta, Sp1, RXR-b, ATF, eta, Sp1, C/EBPdel, GATA-1, Oct-1, T3R-alpha, C/EBPalp, Antp, Fzt, c-Ets-1, AP-1, HNF-1C, C/EBPalp,  GATA-1, CeMyoD, NF-1, Sp1, Sp1, AP-4, MyoD, Oct-1, C/EBPalp, C/EBPalp, AGIE-BP1, NF-kappaB, NF-kappa, Ftz, c-Jun, TBP, Hb, GR, Dl, Oct-1, GATA-1, Oct-1, Oct-1, Odd, GATA-1, ER, Oct-11, C/EBPalp, Zen-1, Pit-1a, C/EBPbeta, Sp1, ICSBP, C/EBPalp, C/EBPalp, HNF-3B, TEC1, GATA-1, TBP, Oct-1, Pit-1a, Antp, Pit-1a, Elf-1, AP-2alph, Oct-1, C/EBPalp, RSRFC4, Ftz, Oct-1, GATA-1, Oct-1, Pit-1a, C/EBPalp, LyF-1, C/EBPalp, C/EBPalp, Oct-1, TBP, Pit-1a, Oct-1, ICSBP, C/EBPalp, MCM1, GATA-1, Erg-1, TBP, TBP, Pit-1a, MEB-1, Oct-1, C/EBPalp, Zen-1, GATA-1, MATalpha2, Oct-1, NF-kappaB, HNF-1C, Oct-1, Oct-1, C/EBPalp, Zen-1, Eve, C/EBPalp, Oct-1, embryo_D, MCM1, MEB-1,C/EBPalp, TBP, TBP, IRF-1, TBP, Oct-1, Net, C/EBPalp, SOX-9, Oct-1, GATA-1, Ftz, TBP, Oct-1, GATA-1, Oct-1, SRF, ER, REV-ErbA, ARP-1, FKBP59, COUP, Oct-1, SRF, Pit-1a, Oct-1, Oct-1, Oct-5, SRF, TBP, Pit-1a, TBP, TBP, TBP, W T1_I_-K, W T1_I-de, Sox-2, WT1_I, WT1-del2, WT1_I_-K, Sox-2, WT1_I, WT1-del2, WT1_I-de, Oct-1, SRF, Ftz, TBP, Pit-1a, Oct-11, Oct-1, TBP, C/EBP, Pit-1a, SRF, TBP, Id3, NF-ATc3, HNF-1, TBP, C/EBPalp, ATF, Adf-1, RAP1, SRF, Odd, RAP1, Sp1, RAP1, RAP1, RAP1, RAP1, RAP1 |
| P38 | *L. vannamei* | 113815719 | 20870106.1 | 179 | HNF-1C, SGF-1, GATA-1, TBP, C/EBP, C/EBPalp, Oct-1, NF-kappaB, C/EBPalp, GATA-1, WT1, Sp1, Oct-1, HNF-3B, TBP, C/EBPalp, C/EBPalp, Hb, C/EBPalpha, C/EBPbeta, HNF-3, GATA-1, Oct-1, AP-1, Sp1, Zen-1, Oct-1, GR, Sp1, NF-1, RAP1, NF-1, NF-1, NF-1, C/EBPalp, Hb, C/EBPalp, C/EBPalp, Antp, CP1, USF, MyoD, NF-1, HNF-1, EBF, GATA-1,Sp1, PU.1, C/EBPalp, CRE-BP1, CPE_bind, Sp1, C/EBPbeta, Oct-1, MEB-1, Ftz, Zen-1, C/EBP, Pit-1a, GCN4, Oct-1, AP-1, GATA-1, NF-kappaB, Oct-1, NF-1, NF-1, Oct-1, C/EBPalpha, C/EBP, C/EBPalp, ICSBP, C/EBPalp, GATA-1, C/EBPalp, C/EBPalp, HNF-1, Id3, NF-AT1, AP-2 c3, GATA-1, Oct-1, HNF-1, Oct-1, ICSBP, NF-1, SRF, YY1, CRE-BP1, ATF, HNF-3, GR, TBP, GR, GATA-1, HOXA7, HNF-1, Oct-1, Ubx, GATA-1, CRE-BP1, CPE_bind, ATF, C/EBPalp, NF-1, TBP, Oct-1, Pit-1a, SRF, MCM1, C/EBPbeta, Oct-1, SOX-9, AP-1, ATF, MATalpha1, Oct-1, C/EBPalp, Hb, Oct-1, YY1, SRF, HNF-3B, TBP, SRF, Oct-1, TBP, C/EBPalpha, Oct-2.1, Ftz, TBP-2, C/EBPalp, Pit-1a, Oct-1, C/EBPalp, GR, Oct-1, SRF, TBP, TBP, TAF-1, Oct-1, C/EBPbeta, AP-1, HNF-3, TBP, PR, TBP, Oct-1, GR, Oct-1, Sp1, Sp1, Oct-1, C/EBPalp, HNF-3, C/EBPalp, Sp1, HNF-3, GR, NF-EM5, C/EBPalp, RAP1, Sp1, WT1_I_-K, Sox-2, WT1_I, WT1-del2, WT1_I-de, RAP1, TBP, Sp1, GAL4, Elf-1, NF-kappa, HNF-3, C/EBPalp, C/EBPeps, C/EBPalp |
| Penaeidin 3a | *L. vannamei* | 113808997 | 20869378.1 | 195 | GR, HSE-bind, PR, GATA-1, myogenin, Sp1, GATA-1, HNF-1C, Oct-2.1, ICSBP, p40x, IRF-1, TEC1, Sox-4, GR, GATA1, Sp1, NF-kappaB, NF-kappa, TBP, TFIID,GR, ICSBP, AP-1, GCN4, GR, TBP, TBP, TBP, TBP, SRF, RAP1, RAPI, SP1, RAP1, GATA-1, SRY, TBP-2, C/EBPbeta, Oct-1, HNF-3, Pit-1a,Hb, C/EBPalpha, Antp, Pit-1a, C/EBPalp, Sp1, C/EBPalp, MCM1, C/EBPalp, C/EBPalp, GCN4, Oct-6, SOX-9, Oct-1, Sp1, SRF, T3R, MyoD, Sp1, REV-ErbA, ER, COUP, AP-1, GATA-1, Erg-1, GATA-1, Oct-1, Pit-1a, Oct-11, Antp, C/EBPalp, Oct-1, Hb, embryo_D, C/EBPalp, TBP, Pit-1a, Ftz, TBP, TBP, TBP, TBP, TBP, TBP, Pit-1a, GATA-1, Ubx, Oct-1, RSRFC9, RAP1, Sp1, SRF, C/EBPalp, GR, ER, NF-kappa, NF-kappaB, T-RAR, T3R, tx, alph, alpha, C/EBPalp, Antp, SGF-3, C/EBPalpha, Pit-1a, Oct-1, C/EBPbeta, C/EBPalp, HNF-1, GATA-1, GR, Pit-1a, GATA-1, C/EBPalp, Oct-1, GATA-1, C/EBPalp, GATA-1, GR,GATA-1, Oct-1, TEF, C/EBPalpha, Zen-1, Pit-1a, GATA-1, Oct-1, p40x, GATA-1, NF-1, GATA-1, C/EBP, Antp, TBP, CPE_bind, Oct-1, TBP, Odd, C/EBPalp, Oct-1, TBP, GCN4, Hb, MIG1, NF-1, YY1, NF-1, Oct-1, NF-kappa, NF-kappaB, GR, CREB, C/EBPalp, CRE-BP1, embryo_D, Sp1, HNF-1, GCN4, Oct-1, SRF, NF-1, Sp1, COUP, C/EBPbeta, Hb, C/EBPalp, GCN4, C/EBPalp, C/EBPalp, Pit-1a, IRF-1, ISGF, C/EBPalp, C/EBPalp, C/EBPalp, GATA-1, Oct-1, NF-1, C/EBPalp, C/EBPalp, ICSBP, Oct-1, C/EBPalp, HNF-3, GR, Pit-1a, NF-1, MEB-1, GLO, Antp, Oct-1, TFIID |
| Phenoloxidase-1 | *L. vannamei* | 113828755 | 20872432.1 | 187 | C/EBP, NF-1, Ftz, Oct-1, HSE-bind, Sp1, AP-1, NF-kappa, Oct-2.1, Oct-1, GR, WT1_I_-K, Sox-2, WT1_I, WT1-del2, WT1_I-de, TBP, MCM1, SRF, C/EBPbeta, Sp1, Sp1, E1, USF, NF-1, NF-kappa, Sp1, USF, C/EBPalp, Oct-1, NF-kappa, NF-kappaB, GCN4, AP-1, c-Fos, c-Jun, Sp1, Sp1, YY1, C/EBPalpha, C/EBPdel, CPE_bind, Zen-1,GATA-1, Oct-1, SRF, Pit-1a, Oct-2.1, Pit-1a, Odd, C/EBPalp, Ubx, GATA-1, Oct-1, TBP, Pit-1a, CPE_bind, GATA-1, Oct-1, Sp1, NF-1, Sp1, MyoD, C/EBPalp, C/EBPalpha, C/EBPalp, SRY, Sp1, ETF, Sp1, Sp1, WT1_I_-K, Sox-2, WT1_I, WT1-del2, WT1_I-de, WT1_I_-K, Sox-2, WT1_I, WT1-del2, WT1_I-de, WT1_I_-K, WT1_I-de, Sox-2, WT1_I, WT1-del2, WT1_I_-K, C/EBPgam, C/EBPalp, Sox-2, WT1_I, WT1-del2, WT1_I-de, WT1_I_-K, Sp1, GATA-1, C/EBPalp, Sp1, E2, HNF-1C, C/EBPalpha, MATalpha1, MCM1, C/EBPalp, RSRFC4, COUP, USF, HNF-3, C/EBPalp, Oct-1, Antp, TBP, ER, T3R-alph, AP-1, Sp1, NF-1, C/EBPalp, TBP, GR, Oct-1, C/EBPalp, C/EBPalp, Oct-1, Hb, C/EBPalpha, NF-ATc3, Oct-1, Antp, HP1_site, HNF-1, C/EBPalp, Elf-1, GATA-1, C/EBPalpha, Oct-1, Oct-1, MEB-1, GLO, C/EBPalp, C/EBPalpha, p40x, TBP, Oct-1, Ftz, C/EBPalp, CREB, CPE_bind, GATA-1, Oct-11, GCN4, C/EBPalp, AP-1, SRF, NF-1, Kr, C/EBPalp, Oct-1, Hb, C/EBPalp, HNF-3, HNF-3, Hb, C/EBPbeta, Sp1, C/EBPalp, TBP, AP-2alph, Sp1, Sp1, Sp1, Sp1, Sp1, Sp1, RXR-beta, Sp1, Sp1, Sp1, Kr, Hb, HNF-3, C/EBPalp, TBP, ICSBP, TBP, Sp1, C/EBPalp |
| Ras-like GTPase | *L. vannamei* | 113809844 | 20869463.1 | 233 | GATA-1, Oct-1, ICSBP, Odd, C/EBPbeta, HNF-3, Oct-1, GCN4, HNF-3B, LyF-1, MEB-1, GLO, C/EBPbeta, Oct-2.1, PR, GR, TBP, C/EBPbeta, Oct-1, TBP, HNF-1, HP1_site, Ftz, GATA-1, MEB-1, GLO, Oct-1, Dl, Odd, Oct-1, TBP, SRY, WT1_I_-K, RAP1, RAP1, Sp1, Sp1, Sox-2, WT1_I_-K, Sox-2, WT1_I, WT1-del2, WT1_I-de, WT1_I_-K, Sox-2, WT1_I, WT1-del2, WT1_I-de, Sp1, WT1_I_-K, Sox-2, WT1_I, WT1-del2, WT1_I-de, RAP1, Sox-2, WT1_I_-K, WT1_I, WT1-del2, WT1_I-de, Oct-1, WT1_I_-K, Sox-2, WT1_I, WT1-del2, WT1_I-de, RAP1, WT1_I_-K, Sox-2, WT1_I, WT1-del2, WT1_I-de, Sp1, Sox-2, RAP1, Sp1, WT1_I_-K, Sox-2, WT1_I, WT1-del2, WT1_I-de, RAP1, WT1_I_-K, Sox-2, WT1_I, WT1-del2, WT1_I-de, YY1, RAP1, Sox-2, RAP1, WT1_I_-K, Sox-2, WT1_I, WT1-del2, WT1_I-de, RAP1, WT1_I_-K, Sox-2, WT1_I, WT1-del2, WT1_I-de, Sp1, RAP1, HNF-3, Sp1, RAP1, Elk-1, WT1_I_-K, Sox-2, WT1_I, WT1-del2, WT1_I-de, Sp1, RAP1, WT1_I_-K, Sox-2, WT1_I, WT1-del2, WT1_I-de, RAP1, WT1_I_-K, Sox-2, WT1_I, WT1-del2, WT1_I-de, Sp1, RAP1, WT1_I_-K, Sox-2, WT1_I, WT1-del2, WT1_I-de, Oct-1, USF, Oct-1, USF, Oct-1, WT1_I_-K, Sox-2, WT1_I, WT1-del2, WT1_I-de, USF, Oct-1, WT1_I_-K, Sox-2, WT1_I, WT1-del2, WT1_I-de, USF, Oct-1, USF,Oct-1, USF, Oct-1, USF, Oct-1, WT1_I_-K, Sox-2, WT1_I, WT1-del2, WT1_I-de, USF, Oct-1, Sp1, RAP1, WT1_I_-K, WT1_I_-K, Sox-2, WT1_I, WT1-del, WT1_I-de, Sp1, RAP1, RAP1, C/EBPalp, GR, C/EBPbeta, Sox-2, GATA-1, Pit-1a, Oct-1, SRF, TBP, Oct-1, AP-2alphaA, Sp1, Sp1, RAP1, TBP, Dl, AP-1, c-Jun, CRE-BP1, ER, ATF, RAP1, ICSBP, C/EBPalp, HNF-3, AP-1, C/EBPalp, HNF-1, GATA-1, Erg-1, YY1, Oct-1, SRF, Odd, GATA-1, ICSBP, Oct-1, ICSBP, Elf-1, C/EBPalp, Hb, Zen-1, C/EBPalp, ICSBP, GR, Sp1, PU.1, C/EBPalp, TBP, C/EBPalp, Ftz, C/EBPalp, GR, PU.1, Sp1, GATA-1, Sp1 |
| Rhodanese domain | *L. vannamei* | 113830416 | 20872637.1 | 223 | C/EBPbeta, Pit-1a, MEB-1, GLO, Ftz, WT1_I_-K, Sox-2, WT1_I, WT1-del2, WT1_I-de, WT1_I_-KTS, Sox-2, WT1_I, WT1-del2, WT1_I-del2, WT1_I_-K, Sox-2, WT1_I, WT1-del2, WT1_I-de, Sox-2, WT1_I, WT1-del2, WT1_I-de, WT1_I_-K, TBP, TBP, TBP, TBP, C/EBPalp, WT1_I_-K, Sox-2, WT1_I, WT1-del2, WT1_I-de, RAP1, TBP, WT1_I_-K, Sox-2, WT1_I, WT1-del2, WT1_I-de,AP-1, TBP, TBP, TBP, TBP, C/EBPalp, TBP, TBP, Oct-1, TBP, Ftz, Oct-11, TBP, TBP, TBP, SRF, Oct-1, Pit-1a, TBP, Oct-1, Ftz, TBP, TBP, Pit-1a, Ftz, TBP, TBP, TBP, TBP, TBP, TBP, Oct-1, Pit-1a, c-Jun, c-Fos, AP-1, NF-E2, Oct-1, TBP, TBP, TBP, TBP, TBP, TBP, HNF-3, C/EBPalp, SOX-9, Oct-1, Kr, C/EBPalp, TBP, C/EBPalp, HSF1_(lo, Sp1, REV-ErbA, C/EBPalp, GATA-1, SRF, GCN4, LyF-1, TEC1, Pit-1a, C/EBPalp, C/EBPalp, C/EBPdel, Id3, HNF-1, NF-ATc3, Oct-1, GATA-1, MIG1, SRF, Oct-1, Dl, TBP, Oct-2.1, Eve, Ftz, C/EBPeps, Sp1, Oct-1, Id3, GR, MIG1, Sp1, SRF, TBP, C/EBPalp, ER, C/EBPbeta, Sp, Hb, Odd, C/EBPalp, Sp1, PU.1, Sp1, GCR1, PU.1, Hb, GATA-1, Sp1, GR, GR, Oct-1, HNF-1, c-Fos, Sp1, Sp1, E1, MyoD, RAR-alph, RXR-alpha, Vmw65, GATA-1, HSF, GATA-1, GATA-1, PU.1, GATA-1, Oct-1, TBP, GATA-1, Oct-1, C/EBPalp, TBP, Oct-1, TBP, TBP, SRF, Oct-1, Pit-1a, TEF, TBP, WT1_I_-K, Sox-2, WT1_I, WT1-del2, WT1_I-de, WT1_I_-K, Sox-2, WT1_I  WT1-del2, WT1_I-de, WT1_I_-K, Sox-2, WT1_I, WT1-del2, WT1_I-de , WT1_I_-K, Sox-2, WT1_I, WT1-del2, WT1_I-de, WT1_I_-K, Sox-2, WT1_I, WT1-del2, WT1_I-de, WT1_I_-KTS, Sox-2, WT1_I, WT1-del2, WT1_I-del2, WT1_I_-K, Sox-2, WT1_I, WT1-del2, WT1_I-de, C/EBPalp, TBP, TBP, TBP, TBP, Oct-1, Pit-1a, WT1_I_-K, Sox-2, WT1_I, WT1-del2, WT1_I-de |
| ROMO-1 | *L. vannamei* | 113814458 | 20869984.1 | 226 | Oct-1, Oct-1,TBP, SRF, Oct-1, NF-1, C/EBPalp, C/EBPbeta, NF-1, CTF-1, C/EBPalpha, Oct-1, TBP, HNF-3, Oct-1, C/EBPalp, TBP, Pit-1a, C/EBPalp, TBP, Pit-1a, Oct-1, HNF-1, C/EBPalp, TBP, CFF, SGF-1, ER, HNF-1C, GATA-1, TBP, HNF-1, AFP1, Oct-1, Dfd, GATA-1, Eve, Oct-1, Oct-1, AP-1, WT1_I_-K, Sox-2, WT1_I, WT1-del2, WT1_I-de, WT1_I_-K, Sox-2, WT1_I, WT1-del2, WT1_I-de,WT1_I_-K, Sox-2, WT1_I, WT1-del2, WT1_I-de, WT1_I_-K, Sox-2, WT1_I, WT1-del2, WT1_I-de, WT1_I_-K, Sox-2, WT1_I, WT1-del2, WT1_I-de, WT1_I_-K, Sox-2, WT1_I, WT1-del2, WT1_I-de, WT1_I_-K, Sox-2, WT1_I, WT1-del2, WT1_I-de, WT1_I_-K, Sox-2, WT1_I, WT1-del2, WT1_I-de, WT1_I_-K, Sox-2, WT1_I, WT1-del, WT1_I-de, WT1_I_-K, Sox-2, WT1_I, WT1-del2, WT1_I-de, WT1_I_-K, Sox-2, WT1_I, WT1-del2, WT1_I-de, WT1_I_-K, Sox-2, WT1_I, WT1-del2, WT1_I-de, WT1_I_-K, Sox-2, WT1_I, WT1-del2, WT1_I-de, WT1_I_-K, Sox-2, WT1_I, WT1-del2, WT1_I-de, SRF, TBP, Oct-1, Dl, GR, NF-kappa, Oct-1, YY1, HNF-1, Hb, CRE-BP1, ICSBP, C/EBPalp, C/EBPdel, C/EBPalp, Oct-1, TBP, C/EBPalp,TBP, GCN4, Hb, HNF-3B, C/EBPalp, Oct-2.1, Hb, Sp1, Egr-1, ETF, C/EBPalp, GATA-1, Sp1, ETF, Sp1, Krox-20, Sp1, C/EBPalp, Oct-1, NF-kappaB, NF-kappaB, Sp1, C/EBPalp, C/EBPdel, NF-EM5, RAR-alph, T3R-alpha, ARP-1, E1, CeMyoD, Sp1, Hb, ICSBP, NF-1, Sp1, C/EBPalp, C/EBPalpha, NF-1, Ftz, AP-2alph, Sp1, YY1, C/EBPalp, REV-ErbA, Sp1, Sp1, Oct-1, T3R, Sp1, Sp1, T3R, c-Jun, AP-1, AP-1, CPC1, c-Fos, Oct-1, TBP, C/EBPbeta, C/EBPdel, Sp1, REV-ErbA, Sp1, Antp, Sp1, Elf-1, Sp1, Sp1, GATA-1, MEB-1, Oct-1, embryo_D, C/EBPalp, TBP, NF-1, SRF, Hb, Sp1, SRF, MCM1, NF-1, Oct-1, C/EBPalp, Sp1, NF-kappaB, AP-1, CRE-BP1, Oct-1, C/EBPalpha, Sp1, AP-2alph, GATA-1, C/EBPalp, C/EBPalp, Fra-2, C/EBPalp, Sp1, USF |
| Serpin | *L. vannamei* | 113821900 | 20870801.1 | 169 | C/EBPalp, Oct-1, GATA-1, MEB-1, NF-kappa, Sp1, PU.1, GCR, TEC1, SRF, CRE-BP1, Oct-1, C/EBPalp, Oct-1A, Hb, Sp1, YY1, CoS, NF-1, AP-1, c-Fos, AP-1, C/EBPgam, Sp1, Oct-1, Egr-1, Sp1, c-Jun, c-Fos, AP-1, AP-1, REB1, C/EBPeps, Sp1, Sp1, CACCC-bi, Sp1, Sp1, Sp1, Sp1, GR, C/EBPgam, GATA-1, Oct-1, Egr-1, Sp1, NF-kappa, HNF-1, C/EBPalp, Krox-20, Sp1, represso, Sp1, REB1, Sp1, GR, TBP, T3R-alpha, Sp1, REV-ErbA, Oct-1, Pit-1a, Oct-1, GATA-1, Oct-1, GR, Oct-1, TBP, Oct-2.1, Pit-1a, GATA-1, C/EBPalp, IRF-1, Antp, Oct-1, Oct-1A, TBP, C/EBPalp, MEB-1, TBP, YY1, SRF, Elf-1, C/EBPalp, Hb, Oct-1, Pit-1a, TBP, GR, C/EBPalp, Oct-1, TBP, Sp1, TBP, TBP, TBP, TBP, TBP, WT1_I_-K, Sox-2, WT1_I, WT1-del2, WT1_I-de, SRF, TBP, RAP1, RAP1, Oct-1, TBP, TBP, TBP, TBP, TBP, Ftz, C/EBPalp, TBP, SRF, Pit-1a, Oct-1, TBP, TBP, Pit-1a, TBP, TBP, TBP, TBP, TBP, TBP, TBP, TBP, Pit-1a, Ftz, TBP, TBP, TBP, TBP, TBP, TBP, SRF, RAP1, RAP1, RAP1, TBP, TBP, TBP, TBP, TBP, TBP, TBP, TBP, TBP, Oct-1, C/EBPalp, TBP, Ftz, Pit-1a, Oct-1, TBP, TBP, TBP, TBP, TBP, TBP, TBP, TBP, GATA-1, GATA-1, TBP, SRF |
| SOCs | *L. vannamei* | 113827830 | 20872191.1 | 247 | Antp, Oct-1, ICSBP, Antp, Oct-1, C/EBPalp, Antp, Ftz, C/EBPalp, Ftz, Antp, MEB-1, GLO, TBP, Oct-1, Antp, C/EBP, GATA-1, Hb, MEB-1, GLO, Oct-11, Pit-1a, C/EBPalp, Antp, Oct-11, Oct-1, SRY, C/EBPalp, Antp, Antp, C/EBP, GATA-1, C/EBPalp, Antp, Antp, Ftz, C/EBPalp, Oct-1, GATA-1, TBP, Oct-1, C/EBPbeta, HNF-1C, GATA-1, NF-1, GATA-1, C/EBPbeta, CRE-BP1, NF-1, Antp, TBP, RAP1, C/EBPalp, CREMdelt, Sp1, RAP1, NF-1, Sp1, Egr-1, Sp1, CPE_bind, ETF, Sp1, Krox-2, Sp1, WT1, ETF, Sp1, Krox-20, C/EBPalp, Oct-2.1, GATA-1, RAP1, Sp1, CACCC-bi, RAP1, Sp1, RAP1, HNF-3, RAP1, RAP1, Sp1, RAP1, RAP1, RAP1, RAP1, RAP1, RAP1, C/EBPalp, Oct-1, Sp1, RAP1, RAP1, HNF-3, GR, TBP, Oct-1, RSRFC4, TBP, Pit-1a, C/EBPalp, TBP, Oct-1A, TBP, Pit-1a, TEC1, Hb, TBP, MATalpha2, Oct-1, Sp1, BRF1, p40x, Oct-1, C/EBPbeta, Sox-2, WT1_I_-K, Sox-2, WT1_I, WT1-del2, WT1_I-de, WT1_I_-KTS, Sox-2, WT1_I, WT1-del2, WT1_I-del2, WT1_I_-K, Sox-2, WT-del2, WT1, WT1_ I-de, WT1_I_-K, Sox-2, WT1_I, WT1-del2, WT1_I-de, WT1_I_-K, Sox-2, WT1_I, WT1-del2, WT1_I-de, WT1_I_-K, Sox-2, WT1_I, WT1-del2, WT1_I-de, WT1_I_-K, Sox-2, WT1_I, WT1-del, WT1_I-de, TSl2, RAP1, SRF, TBP, TBP, MEB-1, TBP, Pit-1a, TBP, TBP, TBP, TBP, Oct-1, Pit-1a, TBP, TBP, TBP, TBP, TBP, TBP, Ftz, TBP, TBP, TBP,TBP, TBP, TBP, Pit-1a, TBP, WT1_I_-K, Sox-2, WT1_I, WT1-del2, WT1_I-de, WT1_I_-KTS, Sox-2, WT1_I, WT1-del2, WT1_I-del2, WT1_I_-K, Sox-2, WT1_I, WT1-del2, WT1_I-de, WT1_I_-K, Sox-2, WT1_I, WT1-del2, WT1_I-de, WT1_I_-K, Sox-2, WT1_I, WT1-del2, WT1_I-de, Sox-2, SRF, TBP, TBP, TBP, MEB-1, Pit-1a, Oct-1, GATA-1, GATA-1, RAP1, HNF-3, Hb, HNF-3, TBP, HNF-3  C/EBPalpha, Hb, GCN4, Sp1, ETF, Sp1, Egr-1, USF, Sp1, Sp1, Sp1, Sp1, Sp1, Sp1, HNF-3, C/EBPalp, HNF-3, C/EBPalp, Hb, Hb, GATA-3, GATA-1, GATA-1, SRF, C/EBPbeta, C/EBPdel |
| SOD | *L. vannamei* | 113823550 | 20871003.1 | 154 | PEA3, RAR-alph, REV-ErbA, HNF-4alp, C/EBPalp, Pit-1a, Oct-1, p40x, C/EBPalp, C/EBPbeta, Oct-1, YY1, GCN4, ICSBP, E2, GATA-1, HNF-1, SRF, MEF-2, TBP, c-Jun, GCN4, Oct-1, LyF-1, TBP, Ftz, Pit-1, C/EBPbeta, Oct-1, Oct-1, HNF-3, C/EBPalp, TBP, GCN4, Oct-2.1, Oct-1A, Ftz, NF-kappa, C/EBPalp, c-Ets-1, MEB-1, GLO, C/EBPalp, Oct-1, NF-ATc, p40x, Oct-1, HSF, Sp1, HSF, TBP, HNF-1C, ISGF-3, Zen-1, Oct-1, C/EBPalp, Oct-1, GR, GR, Hb, C/EBPalp, Oct-1, GATA-1, C/EBPalp, NF-1, C/EBPalp, YY1, GCN4, C/EBPalpha, GATA-1, CRE-BP1, Dfd, RAP1, MEB-1, C/EBPalp, NF-1, embryo_D, Id3, C/EBPalp, c-Jun, AP-1, NF-1, C/EBPgam, Oct-1, Sp1, Sox-2, SRF, TBP, TBP, SRF, RAP1, RAP1, RAP1, Oct-1, RAP1, C/EBPalp, C/EBPalp, SRF, GCR1, TFIID, TBP, Ftz, Pit-1a, C/EBPalp, TBP TBP, RAP1, TBP, USF, RAP1, TBP, Sox-2, WT1_I, WT1-del2, Sox-2, WT1_I-de, WT1_I_-K, WT1_I, WT1-del2, WT1_I-de, SRF, TBP, TBP, Pit-1a, TBP, RAP1, Sp1, RAP1, TBP, Pit-1a, NF-A, Oct-1A, TBP, Oct-1, TBP, Oct-1, TBP, SRF, Oct-1  C/EBPalp, Eve, Id3, HNF-1, NF-ATc3, TBP, TBP, Oct-1, SRF, Oct-1, Pit-1a, TBP, Oct-1, C/EBPalp, p40x |
| Thymosin beta-like | *L. vannamei* | 113827561 | 20872129.1 | 211 | TBP, p40x, C/EBPbeta, RAP1, E4, myogenin, C/EBPalp, Pit-1a, Oct-1, Hb, C/EBPalpha, Zen-1, Oct-1, SOX-9, C/EBPbeta, TBP, HNF-3, C/EBPalp, HOXA4, C/EBPalpha, Oct-1, GATA-1, C/EBPalp, HSE-bind, Oct-1 Antp, GATA-1, SRF, Oct-1, C/EBPalp, Pit-1a, Oct-1, Oct-6, C/EBPalp, TBP, Oct-11, SRF, c-Jun, c-Fos, Pit-1, C/EBPalp, TBP, RAP1, C/EBPalp, C/EBPalp, ICSBP, HNF-1, C/EBPalp, NF-kappaB, Dfd, SRF, TBP, Hb, Pit-1a, HNF-3B, C/EBPalp, Oct-2.1, Oct-1, Oct-1, Ubx, HNF-1, Oct-1, Oct-6, ICSBP, Sp1, Sp1, Antp, C/EBPalp, NF-1, Sp1, C/EBPalp, Sp1, C/EBPalp, Dfd, Pit-1, Oct-11, T3R-alpha, GR, Sp1, GATA-1, Hb, MEB-1, GLO, ICSBP, GATA-1, NF-1, AP-1, JunD, AP-1, Sp1, IPT, RF-1, F1-beta, Sp1, TBP, Sp1, C/EBPalp, NF-1, HNF-1C, C/EBPalp, MATalpha, MCM1, SRF, USF, NF-1, c-Rel, GATA-1, NF-kappaB, RAR-alph, GR, TCF-1alp, CPE_bind, Sp1  Krox-20, SRF, Oct-1, C/EBPalp, Pit-1a, Ttx, ER, RAR-alph, RXR-alpha, AP-1, C/EBPalp, GR, PR, C/EBPalp, MEB-1, GATA-1, TBP, Sp1, TBP, C/EBPalp, GATA-1, MATalpha2, C/EBPalpha, C/EBPalp, GATA-1, Oct-1, CPC, AP-1, RXR-beta, Sp1, Sp1, c-Jun, c-Fos, CP1, GATA-1, ER, LyF-1, GR_alpha, PR, c-Ets-1, REB1, Sp1, C/EBPalp, Sp1, Sp1, C/EBPalp, NF-1, T3R-alpha, GR, MBP-1_(1), c-Rel, EBP-1, NF-kappaB, NF-kappaB, C/EBPalp, REV-ErbA, COUP, RXR-beta, Sp1, Oct-2.1, MEF-2, E2, C/EBPalpha, Sp1, Sp1, ER, Ttx, SRF, MyoD, E1, Sp1, NRL, Oct-1A, C/EBPalpha, TBP, AP-1, Odd, Pit-1, YY1, C/EBPbeta, C/EBPalp, Sp1, Sp1, NF-kappaB, Sp1, NF-1, NF-kappaB, c-Rel, AP-1, NF-E2, C/EBPalp, c-Jun, c-Fos, AP-1, PR, TEC1, Sp1, CACCC-bi, C/EBPalp |
| TGF-β receptor | *L. vannamei* | 113808959 | 20868425.1 | 172 | C/EBP, Oct-1, HNF-1, TBP, Hb, GR, PR, ATF, HNF-3, GCN4, Pit-1b, Oct-11, GATA-1, C/EBPalp, GATA-1, GATA-1, Oct-1, SGF-1, Hb, C/EBPalp, Dl, Sp1, Oct-1, GATA-1, GATA-1, Oct-1, C/EBPdel, NF-kappa, C/EBPalp, SRF, C/EBPdelta, GATA-1, PU.1, TBP, Sp1, c-Ets-1, C/EBPalpha, SRF, MCM1, GATA-1, Sp1, Oct-1A, Elk-1, TBP, TFIID, NF-kappaB, Sp1, Sp1, C/EBPalp, Sp1, ETF, GR, NF-kappaB, Sp1, Sp1, CACCC-bi, Sp1, C/EBPbeta, C/EBPdel, GATA-1, Sp1, Sp1, GATA-1, Sp1, GATA-1, ICSBP, GATA-1, Sp1, Sp1, Sp1, GATA-1, Sp1, Sp1, Sp1, Sp1, AP-2alph, RXR-be, EtaR, Sp1, Sp1, Egr-1, Sp1, NRF-1, NRF-1, WT1, Sp1, Adf-1, Sp1, Sp1, GAL4, NF-kappaB, Sp1, CRE-BP1, CREB, ATF, Sp1, ETF, Sp1, CTF, RAP1, Sp1, Sp1, Egr-1, ETF, Sp1, Sp1, Sp1, RAP1, Sp1, RAP1, RAP1, RAP1, RAP1, RAP1, Sp1, RAP1, RAP1, RAP1, RAP1, RAP1, RAP1, Sp1, Sp1, Sp1, ALF1B, GR, C/EBPalp, Pit-1a, Zen-1, Pit-1a, TEC1, AP-1, Sp1, C/EBPalp, Hb, YY1, C/EBPalp, C/EBPalp, c-Jun, c-Fos, AP-1, Sp1, AP-2alph, CRE-BP1, CREB, Sp1, Oct-1, C/EBPdel, GR, PR, Sp1, ICSBP, ISGF-3, Dfd, GATA-1, C/EBPalp, HNF-3B, C/EBPalp, Pit-1a, Hb, HNF-3B, Oct-1, C/EBP, Sp1, c-Fos, CREB, AP-1, C/EBPalpha, Oct-1, PU.1, Hb, NF-1 |
